# Screening of the antileishmanial and antiplasmodial potential of synthetic 2-arylquinoline analogs

**DOI:** 10.1038/s41598-023-43805-4

**Published:** 2023-10-16

**Authors:** Roger Espinosa-Saez, Sara M. Robledo, Tatiana Pineda, Javier Murillo, César Zúñiga, Osvaldo Yañez, Plinio Cantero-López, Alex Saez-Vega, Camilo Guzmán-Teran

**Affiliations:** 1 Grupo de Investigación, Evaluación y Desarrollo de Fármacos y Afines-IDEFARMA, Departamento de Regencia y Farmacia, Universidad de Córdoba, Montería, Córdoba Colombia; 2 Programa de Estudio y Control de Enfermedades Tropicales-PECET, Facultad de Medicina, Universidad de Antioquia, Medellín, Antioquia Colombia; 3 Corporación de Innovación Para el Desarrollo de Productos, Medellín, Antioquia Colombia; 4 Instituto de Ciencias Naturales, Facultad de Medicina Veterinaria y Agronomía, Universidad de Las Américas, Sede Providencia, Santiago, Chile; 5 Facultad de Ingeniería y Negocios, Universidad de las Américas, Santiago, Chile; 6 Núcleo de Investigación en Data Science, Facultad de Ingeniería y Negocios, Universidad de las Américas, Santiago, Chile; 7 Departamento de Ciencias, Facultad de Ciencias Exactas, Químicas, Universidad Andres Bello, Viña del Mar, Chile; 8 Center of Applied Nanoscience (CANS), Facultad de Ciencias Exactas, Universidad Andres Bello, Santiago, Chile; 9 Relativistic Molecular Physics Group (ReMoPh), PhD Program in Molecular Physical Chemistry, Facultad de Ciencias Exactas, Universidad Andres Bello, Santiago, Chile; 10 Escuela de Ciencias Aplicadas e Ingenierías, Universidad EAFIT, Medellín, Colombia

**Keywords:** Biochemistry, Drug discovery

## Abstract

In this study, six analogs of 2-arylquinoline were synthesized and evaluated for their in vitro and in vivo antiplasmodial and leishmanicidal activity. At a later stage, hemolytic activity and druggability were tested in vitro and in silico, respectively, observing as a result: firstly, compounds showed half-maximal effective concentration (EC_50_) values between 3.6 and 19.3 µM. Likewise, a treatment using the compounds **4a–f** caused improvement in most of the treated hamsters and cured some of them. Regarding the antiplasmodial activity, the compounds showed moderate to high activity, although they did not show hemolytic activity. Furthermore, **4e** and **4f** compounds were not able to control *P. berghei* infection when administered to animal models. Molecular dynamic simulations, molecular docking and ligand binding affinity indicate good characteristics of the studied compounds, which are expected to be active. And lastly, the compounds are absorbable at the hematoencephalic barrier but not in the gastrointestinal tract. In summary, ADMET properties suggest that these molecules may be used as a safe treatment against *Leishmania*.

## Introduction

Leishmaniasis and Malaria belong to a diverse group of parasitic diseases present in countries in tropical and subtropical regions of the world, affecting mainly low-income populations in poor and developing countries^1^ Leishmaniasis has three clinical presentations: cutaneous (CL), visceral (VL), and mucocuatneous leishmaniasis (LM) which are caused by more than 17 species of the *Leishmania* genus, and that transmitted to humans by several species of female Phlebotomus specimens of the *Phlebotomus* and *Lutzomyia* genera^2^. According to the World Health Organization (WHO), Leishmaniasis incidence has been estimated to approximately 12 million cases through all disease presentations and around 350 million people are estimated to live in areas of high-risk of infection. Leishmaniasis mortality has been estimated around 20,000 and 30,000 per year for VL mainly, with 1.5–2 million new cases being reported every year among all clinical presentations. Currently, amphotericin B (AMB), pentamidine isethionate, miltefosine and pentavalent antimonial compounds are being used as antileishmanial drugs for leishmaniasis treatment^1–3^.

Malaria is considered the most threatening human parasitic infection, affecting approximately half of global population and being a motive of concern for centuries. This human disease is caused by five species of parasites belonging to the *Plasmodium* genus, being *P. vivax* and *P. falciparum* the most threatening with and estimated of 247 million cases and 619 deaths in 2021 deaths per year^4^, and it is transmitted to humans through bites of female *Anopheles spp* mosquitoes. Quinine, Chloroquine, Mefloquine, Primaquine and Artemisinin are commonly used antimalarial drugs which have been turning into ineffective malaria treatment due to the increasing prevalence of drug-resistant parasites^4,5^.

Nowadays, drugs used for treating malaria and leishmaniasis represent a limited therapeutic application due to several aspects such as: the highest incidence of disease is presented in marginal areas, where imited drug access, high treatment cost, severe adverse events, emergence of parasitic resistance to treatment, variability in efficacy, high toxicity in pancreas, liver, kidney, and heart associated with prolongated therapeutic regimes with high dosage^6–10^. With that in mind, it is mandatory to continue research for new safe, effective drugs with easy administration and low cost for facilitating its generalized application and contribution to controlling these diseases.

Quinoline nucleus compounds belong to a group of nitrogenated heterocyclic which stand out for their wide range of pharmacological functions^9^, such as antiparasitic activity against protozoans like *P. falciparum*^11^, 4-nitrostyrylquinolines with antiplasmodic activity and excellent selectivity exhibit a cellular action different to current antiplasmodic, acting at the early intraerythrocytic life cycle of malaria parasite, including merozoite invasion with antitrypanosomal and leishmanicidal activities^5,12^. In respect to the latter, 8-aminoquinoline (Sitamaquine) has been considered a potential candidate for oral administration^13^. Likewise, antimalarial based on 8-aminoquinolines such as chloroquine, mefloquine, tanefoquine and primaquine have been reported as effective leishmanicides against several species of *Leishmania*^14^, with quinoline-derived formulations showing promising activity against *L. (V.) panamensis* and *L. braziliensis*^15^. Likewise, 2-styrylquinolines have showed leishmanicidal activity against *L. (V.) panamensis* strains and against in vivo animal infection as well as against *P. falciparum* strains FCB-2 and NF-54^16–18^. Under this scheme, the main objective of this study was to synthesize 2-arylquinoline-like compounds and to evaluate their in silico*, *in vitro and in vivo leishmanicidal, antiplasmodial and hemolytic activities, with aims of developing into new, effective, safe, and easy-administering treatments for CL and Malaria.

## Methods

### Chemicals

All chemicals used for the analysis were analytical grade, Merck and Sigma Aldrich. The Nuclear Magnetic Resonance spectra were obtained by NMR—one-dimensional ^13^C, ^1^H, and DEPT-135; two-dimensional COSY, HSQC, and HMBC—for which a 300 MHz Bruker spectrometer was used (300 MHz for ^1^H and 75 for ^13^C) using Deuterated chloroform (CDCl_3_) as solvent and Tetramethyl Silane [(CH_3_)_4_Si] as an internal standard, chemical shifts (δ) are expressed in parts per million (ppm) and coupling constants (J) in Hertz (Hz). IR spectra were recorded on a Nicolet FTIR spectrometer with diamond ATR. Silica gel 60 (0.063–0.200 mm) was used for column chromatography, and precoated silica gel plates (Merck 60 F254 0.2 mm) were used for thin layer chromatography (TLC) To monitor the progress of the reaction.

### Synthesis of quinolinic derivatives 4a–f

For the chemical synthesis, a solution of quinaldine or 8-hydroxyquinaldine in acetic anhydride was used, the corresponding aromatic aldehyde was added. This solution was brought to reflux for 12–24 h. Upon completion of the reaction, the mixture was allowed to cool to room temperature, then sodium bicarbonate was added. The mixture was extracted with a mixture of petroleum benzine/ethyl acetate. The organic phase was dried over anhydrous sodium sulphate, filtered, and concentrated under reduced pressure. Then, the crude product was purified by column chromatography (CC), using petroleum benzine/ethyl acetate with an increasing polarity gradient as eluent^12,17,19^. The chemical structures of the compounds were corroborated by NMR spectroscopic techniques: in one dimension (^1^H, and ^13^C) and two dimensions (COSY, HSQC, and HMBC), The individual data are described below:

2-[(*E*)-2-(Quinolin-2-yl)ethenyl]phenylacetate (**4a**) was obtained in the form of white crystals with a yield of 40%; M.p 81–85 °C. IR cm^−1^: 3000–3057 (Ar–OH), 1747 (C=O), 1800–2100 (Ar), 1198–1091(C–O), 1612 (C=C). ^1^H NMR (CDCl_3_, 300 MHz): δ 2.43 (s, 3H, CH_3_CO), 7.60 (d, 1H, J = 8.5 Hz, Ar–H_3_), 8.08 (d, 1H, J = 8.5 Hz, Ar–H_4_), 7.79 (d, 1H, J = 8.8 Hz, Ar–H_5_), 7.50 (t, 1H, J = 7.5 Hz, Ar–H_6_), 7.70 (t, 1H, J = 7.6 Hz, Ar–H_7_), 8.13 (d, 1H, J = 8.6 Hz, Ar–H_8_), 7.74 (d, 1H, J = 16.4 Hz, H_1′_), 7.42 (d, 1H, J = 16.4 Hz, H_2′_), 7.28 (d, 1H, J = 9.0 Hz, Ar–H_3″_), 7.81 (dt, 1H, J = 8.3; 10.2 Hz, Ar–H_4″_), 7.34 (dt, 1H, J = 6.1; 6.8 Hz, Ar–H_5″_), 7.14 (dd, 1H, J = 7.8; 7.9 Hz, Ar–H_6″_), ^13^C-NMR (CDCl_3_): δ 21.20 (CH_3_CO), 169.37 (C=O), 155.60 (C-2), 119.2 (C-3), 129.40 (C-4), 129.10 (C-4a), 127.53 (C-5), 126.40 (C-6), 129.80 (C-7), 136.50 (C-8), 148.67 (C-8a), 127.71 (C-1′), 131.23 (C-2′), 127.40 (C-1″), 148.12 (C-2″), 126.3 (C-3″), 127.00 (C-4″), 129.47 (C-5″), 122.9 (C-6″).

2-[(*E*)-2-(8-Hydroxyquinolin-2-yl)ethenyl]phenylacetate (**4b**) was obtained as a yellow solid at a yield 71%; M.p: 120–123 °C. IR cm^−1^: 3200 (OH), 3052 (C–H), 1748 (C=O), 1800- 2100 (Ar), 1182–1077 (C–O), 1614 (C=C); ^1^H NMR (CDCl_3_, 300 MHz): δ 2.42 (s, 3H, CH_3_CO), 7.60 (d, 1H, J = 8.5 Hz, Ar–H_3_), 8.14 (d, 1H, J = 8.6 Hz, Ar–H_4_), 7.79 (d, 1H, J = 7.8 Hz, Ar–H_5_), 7.39 (t, 1H, J = 7.5 Hz, Ar–H_6_), 7.36–7.25 (m, 1H, Ar–H_7_), 7.75 (d, 1H, J = 16.0 Hz, H_1′_), 7.42(d, 1H, J = 16.0 Hz, H_2′_), 7.36–7.25 (m, 1H, Ar–H_3″_), 7.18 (dd, 1H, J = 7.5; 7.6 Hz, Ar–H_4″_), 7.14 (dd, 1H, J = 8.0; 7.8 Hz, Ar–H_5″_), 7.36–7.25 (m, 1H, Ar–H_6″_). ^13^C NMR (CDCl_3_): δ 21.17 (CH_3_CO), 169.26 (C=O), 153.24 (C-2), 120.49 (C-3); 136.80 (C-4), 129.03 (C-4a), 127.17 (C-5); 127.80 (C-6), 151.96 (C-8); 148.76 (C-8a), 127.70 (C1′), 127.70 (C2′), 129.03 (C-1″); 151.96 (C-2″), 126.30 (C-3″), 110.47 (C4″); 122.9 (C5″).

2-Methoxy-6-[(*E*)-2-(quinolin-2-yl)ethenyl]phenylacetate (4**c**) was obtained as a white solid with a yield of 87%. M.p: 90–95 °C, IR cm^−1^: 3238 (OH), 3001–3054 (C–H), 1758 (C=O), 1800–2100 (Ar), 1213–1014 (C–O), 1599–1639 (C=C). ^1^H NMR (CDCl_3_, 300 MHz): δ 2.46 (s, CH_3_CO), 3.87 (s, OCH_3_), 7.67 (d, 1H, J = 8.6 Hz, Ar–H_3_), 8.15 (d, 1H, J = 8.6 Hz, Ar–H_4_), 7.80 (d, 1H, J = 8.1 Hz, Ar–H_5_), 7.52 (t, 1H, J = 7.4 Hz, Ar–H_6_), 7.72 (d, 1H, J = 6.2 Hz, Ar–H_7_), 8.10 (d, 1H, J = 8.6 Hz, Ar–H_8_), 7.74 (d, 1H, J = 16.5 Hz, H_1′_), 7.43 (d, 1H, J = 16.4 Hz, H_2′_), 6.96 (d, 1H, J = 7.9 Hz, Ar–H_4″_), 7.26 (t, 1H, J = 7.9 Hz, Ar–H_5″_), 7.43 (d, 1H, J = 8.0 Hz, Ar–H_6″_). ^13^C NMR (CDCl_3_, 75 MHz): δ (ppm) 20.60 (CH_3_CO), 56.02 (OCH_3_), 168.45 (C=O), 155.64 (C-2), 119.27 (C-3), 136.33 (C-4), 127.41 (C-4a), 127.54 (C-5), 126.31 (C-6), 129.65 (C-7), 129.29 (C-8), 148.15 (C-8a), 127.36 (C-1′), 131.58 (C-2′), 130.38 (C-1″), 138.17 (C-2″), 151.45 (C-3″), 112.07 (C-4″), 126.48 (C-5″), 118.22 (C-6″).

2-Methoxy-6-[(*E*)-2-(8-hydroxyquinolin-2-yl)ethenyl]phenylacetate (**4d**) was obtained as a white solid with a yield of 90%. M.p: 158–160 °C, IR cm^−1^: 2843–3046 (C–H), 1757 (C=O), 1800–2100 (Ar), 1192–1071 (C–O), 1591–1636 (C=C). ^1^H NMR (CDCl_3_, 300 MHz): δ 2.48 (s, 3H, CH_3_CO), 3.90 (s, 3H, OCH_3_), 7.64 (d, 1H, J = 8.6 Hz, Ar–H_3_), 8.15 (d, 1H, J = 8.6 Hz, Ar–H_4_), 7.33 (d, 1H, J = 8.3 Hz, Ar–H_5_), 7.23 (dd, 1H, J = 8.3; 7.5 Hz, Ar–H_6_), 7.47 (d, 1H, J = 8.0 Hz, Ar–H_7_), 7.77 (d, 1H, J = 16.5 Hz, H_1′_), 7.38 (d, 1H, J = 16.5 Hz, H_2′_), 7.45 (d, 1H, J = 8.0 Hz, Ar–H_4″_), 7.28 (t, 1H, J = 8.0 Hz, Ar–H_5″_), 6.99 (d, 1H, J = 8.0 Hz, Ar–H_6″_). ^13^C NMR (CDCl_3_): δ 20.32 (CH_3_CO), 56.18 (OCH_3_), 168.87 (C=O), 153.23 (C-2), 120.35 (C-3), 136.52 (C-4), 130.22 (C-4a), 117.54 (C-5), 110.15 (C-6), 127.38 (C-7), 138.18 (C-8), 151.96 (C-8a), 127.38 (C-1′), 130.72 (C-2′), 127.49 (C-1″), 151.47 (C-2″), 151.47 (C-3″), 118.41 (C-4″), 126.68 (C-5″), 111.90 (C-6″).

4-Bromo-2-[(*E*)-2-(quinolin-2-yl)ethenyl]phenylacetate (**4e**) was obtained as white crystals at a yield of 90%; M.p: 139–140 °C. IR cm^−1^: 1758 (C=O), 1800–2000 (Ar), 3055 (C–H), 1589 (C=C), 1100–1193 (C–O). ^1^H-NMR (CDCl_3_, 300 MHz): δ 2.34 (s, 3H, CH_3_CO), 7.63 (d, 1H, J = 8.8 Hz, Ar–H_3_), 8.01 (d, 1H, J = 9,4 Hz, Ar–H_4_), 7.71 (d, 1H, J = 8.0 Hz, Ar–H_5_), 7.45 (t, 1H, J = 7.1 Hz, Ar–H_6_), 7.50 (d, 1H, J = 8,5 Hz, Ar–H_7_), 8.04 (d, 1H, J = 8.9 Hz, Ar–H_8_), 7.60 (d, 1H, J = 16.3 Hz, H_1′_), 7.28 (d, 1H, J = 16.3 Hz, H_2′_), 6.94 (d, 1H, J = 8,6 Hz, Ar–H_3″_), 7.36 (d, 1H, J = 8.6 Hz, Ar–H_4″_), 7.86 (s, 1H, Ar–H_6″_) ^13^C–NMR (CDCl_3_): δ 20.94 (CH_3_CO), 168.92 (C=O), 154.92 (C-2), 129.35 (C-3), 129.35 (C-4), 129.67 (C-4a), 127.46 (C-5), 126.40 (C-6), 119.53 (C-7), 136.43 (C-8), 148.17 (C-8a), 125.95 (C-1′), 132.17 (C-2′), 119.48 (C-1″), 131.19 (C-2″), 124.54 (C-3″), 131.94 (C-4″), 147.53 (C-5″), 129.68 (C-6″).

2-Ethoxy-4-[(*E*)-2-(quinolin-2-yl)ethenyl]phenylacetate (**4f**) was obtained as white crystals with a yield of 739.4 mg (37%); M.p: 120–123. IR cm^−1^: 1759 (C=O), 1600–2000 (Ar), 3.200 (C–OH), 2960–3052 (C–H), 1636 (C=C). ^1^H-NMR (CDCl_3_, 300 MHz): δ 4.14 (q, 2H_,_ J = 6.9 Hz, OCH_2_CH_3_), 1.42 (t, 3H_,_ J = 6.9 Hz, OCH_2_CH_3_), 2.32 (s, 3H, CH_3_CO), 7.69 (d, 1H, J = 9.0 Hz, Ar–H_3_), 8.07 (d, 1H, J = 9.0 Hz, Ar–H_4_), 7.77 (d, 1H, J = 8.1 Hz, Ar–H_5_), 7.48 (t, 1H, J = 7.5 Hz, Ar–H_6_), 7.70 (t, 1H, J = 8.6 Hz, Ar–H_7_), 8.11 (d, 1H, J = 9.0 Hz, Ar–H_8_), 7.60 (d, 1H, J = 16.0 Hz, H_1′_), 7.36 (d, 1H, J = 16.0 Hz, H_2′_), 7.25 (s, 1H, Ar–H_2″_), 7.17(d, 1H, J = 8.2 Hz, Ar–H_5″_), 7.05 (d, 1H, J = 8.2 Hz, Ar–H_6″_) ^13^C-NMR (CDCl_3_): δ 64.33 (CH_2_), 14.71 (CH_3_), 20.56 (CH_3_CO), 168.93 (C=O), 155.75 (C-2), 119.08 (C-3), 129.10 (C-4), 140.45 (C-4a), 127.51 (C-5), 126.28 (C-6), 129.80 (C-7), 136.48 (C-8), 150.63 (C-8a), 133.84 (C-1′), 129.10 (C-2′), 135.38 (C-1″), 111.34 (C-2″), 148.45 (C-3″), 140.45 (C-4″), 120.48 (C-5″), 122.94 (C-6″).

## Biological activity assays

### Compounds

The compounds were solubilized in 0.5% dimethyl sulfoxide (DMSO; Sigma-Aldrich, St Louis MO, USA). For in vitro assays, a stock solution of 200 μg mL^−1^ was prepared in RPMI-1640 medium (Gibco, Thermo Scientific Inc., Waltham, MA, USA); then, four serial fourfold dilutions equivalent to 100, 25, 12.5, 6.25, and 1.625 μg mL^−1^ were prepared. For Amphotericin B (AMB) used as an assay control for leishmanicidal activity, a stock solution was prepared at 1.0 µg mL^−1^ and four dilutions were tested. With the compounds that showed in vitro activity for *L. (V.) panamensis*, cream formulations were prepared for topical application during the treatment of hamsters with experimental cutaneous leishmaniasis. Shortly, compounds were solubilized in castor oil and then incorporated at a concentration of 1% in a cream base containing water, Sodium methylparaben, sodium propylparaben, monobasic sodium phosphate, cetyl alcohol, light mineral oil, emulium delta, white petrolatum and glycerol monostearate^20–23^.

### Parasite

The *L. (V.) panamensis* strain M/HOM/87/UA140 was used, transfected with the gene for green fluorescent protein (UA140-EGFP) generated in a previous work. Parasites were cultured as promastigotes in Novy-MacNeil-Nicolle (NNN) biphasic medium and phosphate buffered saline (PBS) with glucose (pH 6.9) as the liquid phase. The cultures were incubated at 26 °C and maintained as promastigotes. To ensure a greater infection of macrophages in vitro, the *L. (V.) panamensis* strain was maintained by successive passages in experimentally infected hamsters (*Mesocricetus auratus*), making periodic aspirations of hamster lesions using PBS and #26 needles. The aspirated samples were grown in NNN culture medium at 26 °C, until promastigotes were obtained^20–23^.

The *P. falciparum* (3D7 strain) was maintained as asynchronous culture under standard conditions in RPMI-1640 medium enriched with 3% SFB and 1% erythrocytes at 37 °C in 5% O_2_, 5% CO_2_ and 90% N_2_ atmosphere^24^. For in vivo antiplasmodial activity assays, was used the chloroquine sensitive ANKA strain of *P. berghei* (generously donated by Dr. S Blair, Universidad de Antioquia, Medellín-Colombia), as cryo-frozen stock of parasitized red blood cells (PRBCs). The parasites were prepared through two cycles of passage of the PRBCs in three BALB/c mice injected intraperitoneally (ip) with 100 µL of PRBCs as describe elsewhere^25^*.*

### Cells

Human monocytes of the U-937 line (ATCC-CRL-1593-2TM) were kept cultured in suspension in “complete” RPMI-1640 medium—that is, supplemented with 10% fetal bovine serum (FBS; Invitrogen) and a 1% mixture of antibiotics composed of penicillin (10,000 U mL^−1^) and streptomycin (10,000 U mL^−1^; Sigma)—and incubated at 37 °C with 5% CO_2_^20^. Human red blood cells (huRBC) were obtained from the blood Alma Mater Hospital´s blood bank in Medellín, Colombia.

### Cytotoxicity in U-937 macrophages

Cytotoxicity was evaluated in U-937 cells using the enzymatic micromethod with 3-(4,5-dimethylthiazol-2-yl)-2,5-diphenyltetrazolium (MTT) bromide (Sigma). Cells in exponential phase of growth were adjusted to a concentration of 100,000 cells mL^−1^ of complete RPMI-1640 medium. In each well of a 96-well cell culture dish (Falcon, Fisher Scientific, Thermo Scientific Inc., Waltham, MA, USA), 100 µL of cells were deposited. Then, 100 µL of each of the corresponding concentrations of each compound (200, 50, 12.5, or 3.125 µg mL^−1^) was added to each well. As a viability control (no cytotoxicity), cells incubated in complete RPMI-1640 medium were used, while cells exposed to Doxorubicin (DOX) were used as a cytotoxicity control^20^.

The cells in the presence of the different solutions of the compounds at the respective concentrations, as well as the cells exposed to AMB and doxorubicin and those not exposed, were incubated at 37 °C in a 5% CO_2_ atmosphere for 72 h. After the incubation period, 10 µL well^−1^ of a MTT solution with a concentration of 5 µg mL^−1^ (Sigma) was added and the dishes were incubated at 37 °C for 3 h. After this incubation period, 100 μL well^−1^ of a solution of 50% isopropanol (Merck Millipore) and 10% sodium dodecyl sulphate (SDS) (Merck Millipore) was added, in order to solubilize the formazan crystals formed. The plates were incubated for another 30 min and the production of formazan (which is proportional to the percentage of viable cells) was measured in a microplate reader (Benchmark Bio-Rad Hercules, CA, USA) at a wavelength of 570 nm^20^.

Cytotoxicity was determined according to the percentage decrease in the number of living cells obtained for each concentration of compound or AMB, according to the optical densities (OD) obtained in each experimental condition and, in comparison, with the OD obtained from cells not exposed to any compound. The viability of cells after treatment was calculated using the OD values for each evaluated condition, through the following equation: % Viability = [OD cells exposed to the compound ÷ OD cells not exposed] × 100. The OD values obtained for cells in the absence of compounds corresponded to 100% viability^20,21^. Then, the percentage of mortality (also called inhibition of cell growth) was calculated through the following equation: % Mortality = 100 − % Viability.

Finally, with the mortality percentages, the mean lethal concentration (LC_50_) was calculated, using the dose response analysis method Probit with the SAS Data Analysis statistical program (SAS Institute Cary NC, USA). The tests were carried out twice, with three replicates for each concentration evaluated. The cytotoxicity of each compound was classified according to the LC_50_ values as: high when LC_50_ was < 100 µg mL^−1^; moderate when LC_50_ was > 100 but < 200 μg mL^−1^; and low when LC_50_ was > 200 μg mL^−1^^20,23^.

### In vitro antileishmanial activity of compounds 4a–f

The activity of the compounds was evaluated in intracellular amastigotes of *L. (V.) panamensis* obtained after in vitro infection of macrophages U-937. For this, the U-937 cells maintained in suspension culture were centrifuged at 1500 rpm for 10 min and, after discarding the supernatant, the button cells were resuspended at a concentration of 1 × 10^5^ cells mL^−1^ of complete RPMI 1640 medium and 0.1 μg mL^−1^ phorbol myristate acetate (PMA; Sigma). In each well of a 24-well cell culture plate (Falcon), 1 mL of the cell suspension was dispersed and incubated at a temperature of 37 °C with a 5% CO_2_ atmosphere. After 72 h, cells were infected with promastigotes in stationary phase of growth at a ratio of 15:1 parasites cell^−1^ to ensure infection percentages higher than 50%. The dishes were incubated at 34 °C under 5% CO_2_ for 2 h. Subsequently, two washes with PBS were carried out to eliminate free parasites, 1 mL of complete RPMI 1640 medium was added, and the cells were incubated again for 24 h. After this, the infected cells were exposed to each of the concentrations of the compounds for 48 h. As infection control, infected and cultured cells were used in the absence of the compounds and AMB was used as a control of antileishmanial activity. After 48 h of incubation at 34 °C and 5% CO_2_, the cells were carefully removed from the bottom of the plate using trypsin and the plunger of a syringe and analysed in an Argon laser flow cytometer, reading at 488 nm excitation and 525 nm emission (Beckman-Coulter Cytomics^tm^ MCL FC 500 Brea, CA, USA). Each concentration of the compounds, including AMB, was evaluated in triplicate in two different experiments. The antileishmanial activity was determined according to the number of parasites in the infected cells obtained for each concentration of each compound and for AMB, according to the number of positive events for green fluorescence using dot diagrams, as well as according to the average intensity of fluorescence (IFM) using histograms^21^.

The inhibition of infection—that is, the decrease in the number of parasites due to the effect of the evaluated compounds—was calculated using the following equation: % Infection = [MFI infected and exposed cells ÷ MFI infected and unexposed cells] × 100, where the MFI values ​​obtained for infected cells in the absence of compounds corresponded to 100% infection. In turn, the percentage of infection inhibition corresponded to the reciprocal of the infection. With the % inhibition of the infection, the mean effective concentration (EC_50_) calculated by the Probit method was determined, which corresponds to the maximum concentration that reduces 50% of infection. Leishmanicidal activity was classified, according to the EC_50_ values, ​​as follows: high when EC_50_ was < 25 μg mL^−1^; moderate when EC_50_ was > 25 but < 50 μg mL^−1^; and low when EC_50_ > 50 μg mL^−1^. Finally, the cytotoxic activity was correlated with the antileishmanial activity by calculating the Selectivity Index (IS), using the following equation: IS = LC_50_ ÷ EC_50_^26,27^.

### In vivo therapeutic response of 4a–f in hamsters infected with *L. (V.) panamensis*

Juvenile hamsters (6 weeks-old), both sexes, were injected intradermally with 1 × 10^8^ stationary growth phase promastigotes of *L. (V.) panamensis* in 100 μL phosphate buffer saline (PBS), in the dorsum. After development of an ulcer, hamsters were distributed into four experimental groups (n = 5 each). Three groups of hamsters were treated with 40 µL day^−1^ of 1% formulations in castor oil of **4a, 4b, 4c, 4d, 4e** and **4f,** for 20 days. The fourth group of hamsters was treated with intralesional meglumine antimoniate (MA; 200 μg mL^−1^) administered twice per week for 3 weeks. Animal welfare was supervised daily during the study. The size of the lesion was measured at the end of treatment (TD20), as well as at days 30, 60, and 90 post-treatment, denoted by PTD30, PTD60, and PTD90, respectively^23,27^. During these time points, hamsters were weighed and supervised for changes in behaviour, water, and food consumption, as well as the appearance of urine and faeces. In vivo methods used in this research were reviewed and approved by Universidad de Antioquia’s Ethics Committee for Animal Experimentation in Act N°131 of February 11, 2020. Euthanasia of hamster specimens was carried out by sodium pentobarbital overdose. Euthanasia, sample collection and handling of the animals was done in compliance of Center for Diseases Control and Prevention guidelines for Safe Work Practices in Human and Animal Medical Diagnostic Laboratories and ARRIVE guidelines^28,29^.

The effectiveness of each treatment was determined after comparing the lesion sizes prior to and after treatments. The size of the lesions was measured with a digital caliper by measuring the diameter of the lesion and calculating the lesion area. Treatment outcome at the end of study was recorded as *cure* (healing of 100% of the area and complete disappearance of the lesion), *improvement* (any percentage of reduction of lesion), *failure* (low decrease or an increase in the size of the lesion), or *relapse* (reactivation of lesion after initial cure). Toxicity of the topical cream was determined according to changes in the body weight obtained during and after treatment, as well as the levels of alanine amino transferase (ALT), blood urea nitrogen (BUN), and creatinine metabolites in serum before treatment (TD0) and at day 8 of treatment (TD8)^30^.

### In vitro antiplasmodial and hemolytic activity

The effect of the compounds on *P. falciparum* (Strain 3D7) asynchronic cultures was determined by quantification of lactate-dehydrogenase activity (LDH) releases from the cytosol of damaged cell to the supernatant which is an indicator of compound-induced parasite mortality. Briefly, non-synchronized *P. falciparum* cultures were adjusted to a parasitemia of 0.5 and 1% hematocrite in RPMI medium enriched with lipid-rich bovine-serum albumin (3%)–Albumax II. Then 100 µL of parasite suspension were added to each well of a 96-well plate, which were later exposed to 100 µL od each dilution of the compounds (4 serial dilutions of 100, 26, 6.25 and 1.56 µg mL^−1^). Chloroquine (CQ) was used as an antiplasmodial drug positive control. Parasites that were not exposed to any compounds were used as a both a growth and viability control (negative control). Plates were incubated for 48h at 37 °C in gases atmosphere N_2_ (90%), CO_2_ (5%) and O_2_ (5%) m and parasites were collected after incubation to be subjected to three freeze–thaw cycles of 20 min each. In the meantime, 100 µL of Malstat reagent was prepared (400 µL Triton X-100 in 80 mL of H_2_O, 4g lactate, 1.32 g Tris buffer and 0.022 g of acetylpyridine dinucleotide in 200 mL of _dd_H_2_O, adjusted to pH 9.0) and 25 µL o NBT^31–33^.

100 µL of PSA solution (0.16 g NBT salt and 0.08 g of phenazine ethosulphate in 100 mL _dd_H_2_O were added to each well of a 96-well plate. After freeze–thaw cycles, 15 µL were withdrawn from each well and added to the corresponding well of an additional plate (containing Malstat and NBT/PES). After an hour of incubating in the dark and room temperature, LDH colorimetric reaction development was detected at 650 nm in a spectrofluorometer. Color intensity in each experimental condition is registered as optical density (OD). Non-specific absorbance was corrected by subtracting blank’s OD (Malstat and NBT/PES). OD determinations were carried out in triplicates in at least two independent experiments^32,33^.

### Hemolytic activity

Hemolysis inducing capacity was assessed following the spectrophotometry cytotoxicity method in 96-well plates. HuRBC were adjusted to 5% hematocrit in RPMI-1640 medium. Then, 500 µL were added to each well of a 24-well cell culture plate and red blood cells were exposed to 200 µg mL^−1^ of each compound. Detection of free hemoglobin after 48 h of incubation at 37 °C evidences compound-induced hemolysis. Free hemoglobin concentration was measured by 542 nm spectrophotometry and color intensity (absorbance) is registered as Optical Density (OD). Non-specific absorbance was corrected by subtracting blank’s OD value (culture medium). Measures where done in triplicated in at least two independent experiments^32,33^.

### In vivo therapeutic response of 4e and 4f in BALB/c mice infected with *P. berghei*

The antiplasmodial activity of compounds **4e** and **4f** in comparison to chloroquine was evaluated on established infection using the Rane´s test described by Ryley and Peters. Briefly, twenty-four BALB/c mice, 8 weeks-old, both sexes, were infected with 1 × 10^7^ *P. berghei* PRBCs by ip injection on the day 0 (D0). Everyday blood samples from the tail were taken to monitor the parasitemia level in Giemsa-stained thin smears. Seventy-two to 96 h later (D3/D4) when parasitemia was established (5–6% parasitemia), the mice were randomly divided into four groups (n = 6). Two groups of mice were treated orally with 100 μL of 200 mg kg^−1^ day^−1^ **4e** or **4f** for 7 days; a third group of mice was treated with 15 mg kg^−1^ day^−1^ chloroquine (CQ) for 4 days. The negative control group was treated with PBS (100 μL day^−1^), for 7 days. Daily clinical follow-up was performed from the beginning of the treatment until the end point of study (2 days after treatment ended), recording weight, hydration status, alertness, fur appearance, breathing status, reaction to stimuli, isolation, hematuria, among others. Giemsa-stained thin smears were prepared from tail blood samples collected on each day of treatment and at the end of the study (day 2 post-treatment) to monitor parasitemia level by counting 4 fields of approximately 2000 erythrocytes per slide. At the end point of the study mice were euthanized in a CO_2_ chamber and necropsy was performed^32^. Toxicity of treatment was also determined according to changes in the body weight and levels of ALT, BUN, and creatinine in serum before treatment (TD0) and 2 days after the end of treatment (PTD2) which corresponded to the t the end of the study.

## Computational analysis

### Molecular docking and ligand efficiency approach

Molecular docking analyses were performed to study the possible binding modes of **4a–f** to *L. major* dihydroorotate dehydrogenase (*Lm*DHODH)^34,35^ and *L. major* tryparedoxin peroxidase I (*Lm*TXNPx)^36^, as potential inhibitors, as there have been interesting studies of molecular docking of quinoline derivatives, including studies of leishmanicidal activity^37^. Based on the binding energy landscapes, compounds with the most negative scores, and previously published data^38^, these two crystals from *L. major* were prioritized for in vitro validation. The binding sites of *Lm*DHODH and *Lm*TXNPx inhibitors have been characterized, based on structural information derived from the position of the ligand for proteins co-crystallized with a bound ligand. Literature data^35,36,38,39^ were used to determine active site residues for these structures. AutoDock (v 4.2.1) and AutoDock Vina (v 1.0.2)^40^ were used for all dockings in this study. The three-dimensional coordinates of structures **4a–f** structures were obtained from the PubChem database and energy optimized using the MOPAC2016^41^ software, through the PM6-D3H4 semi-empirical method^42,43^. When ligand structures were not available from PubChem, they were drawn using Discovery Studio 3.1 (Accelrys, CA). The ligand files were prepared using the AutoDockTools package^44^, provided by AutoDock, accepting all rotatable bonds. The partial charges of each ligand were determined using the PM6-D3H4 semi-empirical method; this approach introduces dispersion and hydrogen-bonded corrections to the PM6 method. The crystal structures of *Lm*DHODH (PDB Code: 3MJY) and *Lm*TXNPx (PDB Code: 4K1F) were downloaded from the Protein Data Bank^45^. *Lm*DHODH and *Lm*TXNP were treated with Schrödinger's Protein Preparation Wizard^46^; polar hydrogen atoms were added, non-polar hydrogen atoms were merged, and charges were assigned. Docking was treated as rigid and carried out using the empirical free energy function and the Lamarckian Genetic Algorithm provided by AutoDock Vina^47^. The grid map dimensions were 20 × 20 × 20 Å^3^, with 0.375 Å spacing between grid points, making the binding pocket of *Lm*DHODH the centre of the flavin mononucleotide cofactor and S2 subsite catalytic residues (Ser100, Asn128, Gln139, Val140), while the active site of *Lm*TXNP was defined as the centre of Arg128. All other parameters were set as the defaults defined by AutoDock Vina. Dockings were repeated 20 times, with the space search exhaustiveness set to 20.

The best interaction binding energy (kcal mol^−1^) was selected for evaluation. Docking result 3D representations were used, from the Discovery Studio 68 molecular graphics system. In this context, and based on our past experience^17^, we demonstrated that, in *LmDHODH*, the S2 sub-site is essential for the activity of the *LmDHODH* enzyme, which contains active amino acids within the loop (a4–bA)^34,35^. In the case of *LmTXNPx* (PDB Code: 3TUE)*,* catalyse TS2-dependent peroxide detoxification. These types of mechanisms are useful for the design of new drugs, as they are unique to the parasite and necessary for its survival^37,39^. The active site was visualized through PDB Code: 4K1F.

Ligand efficiency (LE) calculations were performed using one parameter, K_d_. The K_d_ parameter corresponds to the dissociation constant between a ligand/protein, and its value indicates the bond strength between the ligand/protein^48–50^ K_d_ calculations were carried out using the following equations:

1 $$\Delta {G}^{0}=-2.303RTlog\left({K}_{d}\right),$$

2 $${K}_{d}={10}^{\frac{\Delta {G}^{0}}{2.303RT}},$$

where ∆G^0^ is the binding energy (kcal mol^−1^) obtained from docking experiments, R is the gas constant, and T is the temperature (in Kelvin). We considered standard conditions of aqueous solution at 298.15 K, neutral pH, and remaining concentrations of 1 M. The LE allows us to compare molecules, according to their average binding energy^50,51^. Thus, it is determined as the ratio of binding energy per non-hydrogen atom, as follows^48,49,52^:

3 $$LE=-\frac{2.303RT}{HAC}\mathrm{log}\left({K}_{d}\right),$$

where K_d_ is obtained from Eq. (2) and HAC denotes the heavy atom count (i.e., the number of non-hydrogen atoms) in a ligand. The obtained results are shown in Tables 5 and 6 and Figures S4 and S5 (Supplementary Material).

### ADMET properties

The goal of calculating ADMET profiles is to provide, with reasonable accuracy, a preliminary prediction of the in vivo behaviour of a compound, in order to assess its potential to become a drug^53^. The molecules used in this study were submitted to the calculation of their absorption, distribution, metabolism, excretion, and toxicological properties (ADMET). Furthermore, physicochemical properties, such as molecular hydrogen bond acceptor (HBA), hydrogen bond donor (HBD), weight (MW), topological polar surface area (TPSA), rotatable bond count (RB), octanol/water partition coefficient (LogP), and Molar Refractivity (MR) were calculated, using the SwissADME webserver^54^. Compound toxicological properties were analysed taking into account the Lipinski, Ghose, Veber, and Pfizer toxicity empirical rules (see Table S1).

### Molecular dynamic simulations

The molecular docking results allowed us to recognize that the LmDHODH–ligand complexes showed more favourable interactions and ligand efficiency. Therefore, the finally selected position of the ligands, based on the docking score and predicted binding energy, was studied to describe the molecular interactions of LmDHODH with the bound ligands over time. In this context, molecular dynamics (MD) simulation showed the dynamic behaviour of the LmDHODH–ligand molecular system, assessing the stability of the complex. The most highly stable conformations for the LmDHODH system were used for the molecular dynamics study with the CHARMM force field. Thus, six LmDHODH complexes were built for each model, where each model was confined inside a periodic simulation box. The water model TIP3P^55^, with 12.552 molecules, was used as solvent. Furthermore, Na^+^ and Cl^−^ ions were added, in order to neutralize the systems and maintain an ionic concentration of 0.15 mol L^−1^. The full geometric optimizations of the two molecules were carried out with the density functional theory method by M05-2X-D3^56,57^, in conjunction with the 6-31G(d,p) basis set. The compounds (**4a**–**f**) and flavin mononucleotide (FMN) were parametrized using the LigParGen web server and the OPLS-AA/1.14*CM1A(-LBCC) force field parameters were implemented for organic ligands^58–60^. The partial charges of each ligand were determined and generated by the restrained electrostatic potential (RESP) model^61^. MD simulations were carried out using the modelled CHARMM22 and CHARMM36 force fields^62–64^. First, each system included 20,000 steps of conjugate-gradient energy minimization, followed by 5 ns of simulation with fixed protein backbone atoms, gradually releasing the backbone over 100,000 ps with 10–0.0 kcal mol^−1^ Å^−2^ restraints. The total duration of simulation was ~ 70 ns for each system. During the molecular dynamic’s simulations, motion equations were integrated with 2 femtoseconds time step in the NPT ensemble at a pressure of 1 atm. SHAKE algorithm was applied to all hydrogen atoms, and Van der Waals cut-off was set to 12 Armstrong. Temperature was maintained at 310 K, employing the Nosé–Hoover thermostat method with a relaxation time of 1 ps. Pressure was controlled at 1 atm by using a Nosé–Hoover–Langevin piston. Long-range electrostatic forces were considered by the particle-mesh Ewald approach. Data was collected every 1 ps during MD runs. Molecular visualization of the systems and MD trajectory analysis was carried out using the VMD software package^65^.

### Free energy calculation

The molecular mechanics with generalised Born and surface area solvation (MM/GBSA) method was employed, in order to estimate the binding free energy of the LmDHODH complexes. For calculations from a total of 70 ns of MD, the last 50 ns were extracted for analysis, and the explicit water molecules and ions were removed. The MM/GBSA analysis was performed on three subsets of each system: the protein alone, the ligand alone, and the complex (protein–ligand). For each of these subsets, the total free energy (${\Delta G}_{tot}$) was calculated as follows:

4 $${\Delta G}_{tot}={E}_{MM}+{G}_{solv}-{T\Delta S}_{conf},$$

where ${E}_{MM}$ is the bonded and Lennard–Jones energy terms, ${G}_{solv}$ is the polar contribution of solvation energy and non-polar contribution to the solvation energy, T is the temperature, and ${\Delta S}_{conf}$ corresponds to the conformational entropy^66^. Both ${E}_{MM}$ and ${G}_{solv}$ were calculated using the NAMD software with the generalized Born implicit solvent model^67,68^. ${\Delta G}_{tot}$ was calculated as a linear function of the solvent-accessible surface area, which was calculated with a probe radius of 1.4 Å^69^. The binding free energy of LmDHODH and ligand complexes (${\Delta G}_{bind}$) were calculated by the difference, where ${\Delta G}_{tot}$ values are the averages over the simulation:

5 $${\Delta G}_{bind}={\Delta G}_{tot}\left(complex\right)-{\Delta G}_{tot}\left(Lm\mathrm{DHODH}\right)-{\Delta G}_{tot}\left(ligand\right).$$

### Data analysis

The results are reported as mean values ± standard deviation. The LC_50_, HA_50,_ and the EC_50_ were calculated by Probit analysis using Graph pad Prism 8.0 (San Diego, CA, USA). Since biological activities are defined according to ranges of activity and not according to absolute values, the cytotoxicity and hemolytic activity was defined in terms of high, moderate or low according to activity ranges as follows: high for LC_50_ or HA_50_ values < 100 µg mL^−1^, moderate: for values > 100 µg mL^−1^ but < 200 µg mL^−1^, and low for values < 200 µg mL^−1^. Similarly, the leishmanicidal and antiplasmodial activity of compounds was defined in terms of high, moderate or low according to activity ranges between 0 and 25 µg mL^−1^ for high activity, > 25 µg mL^−1^ but < 50 µg mL^−1^ as moderate activity and < 50 µg mL^−1^ as low activity. Finally, the cytotoxic activity in U937 macrophages was correlated with the leishmanicidal activity by calculating the Selectivity Index (SI), using the following equation: SI = LC_50_ ÷ EC_50_ for *L. (V.) panamensis* while the hemolytic activity in huRBC was correlated with the antiplasmodial activity by calculating the IS using the following equation: SI = HA_50_ ÷ EC_50_ for *P. falciparum*^26,27^.

## Results and discussion

### Chemistry

**Figure 1:**
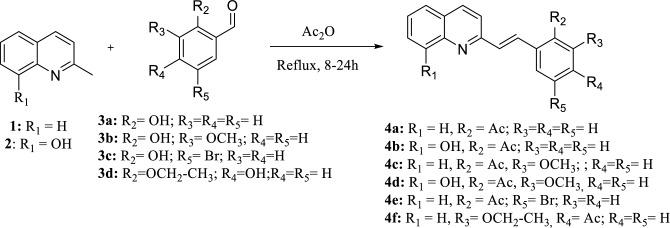
Synthesis of quinoline analogs 4a–f.

Compounds **4a–f**, were obtained using acetic anhydride and high temperatures as a reaction condition, with yields between 37 and 90%. The synthesis strategy was based on the modification of the aromatic aldehyde and the quinolinic ring, to determine the change in biological activity of the compounds (Fig. 1)^17,70^. Chemical structures where confirmed using IR and 1D and 2D RMN techniques, where chemical displacements distinctive of reaction products were recognized with the double bonds with geometric *trans* isomerism of approximately δ 7.74 and 7.43 as doubles with coupling constants of J = 16.4 Hz.

Considering that a compound can be considered cytotoxic when its LC_50_ is achieved at concentrations below 100 µg mL^−1^, compounds **4a–f** showed high cytotoxicity for U-937 cells with LC_50_ values ranging between 9.2 and 25.3 µg mL^−1^ (see Table 1). As expected, AMB, DOX and CQ showed also high cytotoxicity with LC_50_ values of 45.4 µg mL^−1^, 1.2 µg mL^−1^ and 50 µg mL^−1^ respectively. On the other hand, all compounds demonstrated very high activity against intracellular amastigotes of *L. (V.) panamensis,* with EC_50_ < 10 µg mL^−1^ (Table 1). AMB, used as an assay positive control showed high activity (EC_50_ < 1.0 µg mL^−1^) (see Table 1). Nonetheless, when correlating cytotoxicity with leishmanicidal activity, it was observed that compounds **4a–f** yielded values of SI between 3.6 and 15.2 which means that are required 3–15 times more concentrations of the compounds to kill the host cell (macrophages in this case) than to kill the *Leishmania* amastigotes.

**Table 1:** Results of in vitro cytotoxicity and leishmanicidal, antiplasmodial and hemolytic activities of Compounds **4a–f**.

Compounds	Cytotoxicity^a^	Leishmanicidal activity		HA_50_^d^	Antiplasmodial activity	
		CE_50_^b^	IS^c^		CE_50_^b^	IS^e^
4a	8.9 ± 1.8 (30.8)	1.5 ± 0.4 (5.1)	5.9	> 200 (691.2)	20.9 ± 4.3 (72.3)	> 9.6
4b	16.7 ± 3.8 (54.6)	1.1 ± 0.1 (3.6)	15.2	> 200 (655)	29.1 ± 4.4 (95.1)	> 6.9
4c	9.2 ± 1.9 (28.8)	1.5 ± 0.3 (4.7)	6.1	> 200 (626.3)	34.9 ± 2.3 (109)	> 5.7
4d	16.3 ± 3.5 (48.5)	2.4 ± 0.1 (7.1)	6.8	> 200 (596.4)	27.4 ± 6.8 (81.5)	> 7.3
4e	25.3 ± 2.7 (68.8)	7.1 + 0.6 (19.3)	3.6	> 200 (543.1)	17.3 ± 1.4 (47)	> 11.6
4f.	18.4 ± 0.8 (55.3)	2.8 + 0.2 (8.4)	6.6	> 200 (600.3)	16.9 ± 0.7 (50.7)	> 11.8
Amphotericin B (AMB)	45.4 ± 7.8 (49.1)	0.05 ± 0.01 (0.054)	908	NA	NA	NA
Doxorubicin (DOX)	1.2 ± 0.07, 0.02	NA	NA	NA	NA	NA
Chloroquine (CQ)	50 ± 0.0 (156.2)	NA	NA	> 200	3.4 ± 0.4 (10.6)	> 58.8

The results are reported as mean values ± standard deviation. ^a^LC_50_: median lethal concentration in µg mL^−1^ (µM) in U937 macrophages; ^b^EC_50_: median effective concentration in µg mL^−1^ (µM); ^c^Index of selectivity for human macrophages vs *Leishmania* spp (IS) = LC_50_/EC_50._^d^HA_50_: median hemolysis concentration in µg mL^−1^ (µM)); IS for huRBC vs *P. falciparum* IS = HA_50_/EC_50_; ^f^NA: not applicable.

Although a certain degree of activity (toxicity) against the host cell was observed, this is 15 times less than the activity against the parasite inside the cell, suggesting that the required concentration to eliminate 50% of the parasite population is much lower than the required to eliminate 50% of the cell population. On the other hand, it is important to consider that in vitro systems using cells are not definitive for establishing the real toxicity of the compound and defining a possible route of administration of a drug, as it is necessary to carry out tests in an in vivo model that allows parallel measurements of both the properties of absorption, distribution, metabolism and excretion and the toxicity associated with the tissues reached by the compound during its kinetics in the living organism and the effect it causes on the functions of vital organs, especially the liver and kidney. Therefore, the results obtained in studies evaluating toxicokinetics in combination with physicochemical and biopharmaceutical properties determine the most appropriate route of administration for a specific molecule^71,72^.

When comparing the structure of compounds **4a–f** and the leishmanicidal activity in vitro and in vivo, it was observed that the presence of oxygenated substituents (–OH, acetoxy, OMe) in position 8 of the quinoline ring and in positions 2, 3 and 4 of the aldehyde ring could be related to the activity^16,17^. On the contrary, the addition of halogen substituents in the molecule (**4e**) preserves the activity but generates a slight decrease in it. Considering the structure of this type of molecules and, in accordance with the results of previous studies, it could be said that the presence of the styryl group in position 2 in the quinoline ring and oxygenated substituents in the aldehyde and quinoline rings would contribute in the activity. Likewise, structural conditions that contribute to the biological activity of this type of compounds are reported, such as: having an unsaturated structure, an alkyl chain length of no more than 5 carbons and the presence of functional groups in the substituents without shared electrons^12,16,17,73,74^. Furthermore, it has been shown that quinoline derivatives show antileishmanial activity through causing mitochondrial oxidative stress to the parasite^2^.

Although toxicity is an important criterion in the development of new drugs, the specific activity against *Leishmania* parasites favours selecting a drug candidate, as toxicity is a factor that can be managed or reduced through the use of drug delivery systems, such as liposomes, and other types of nano systems that can enhance the pharmacological properties, without loss of the pharmacological potential of the drug candidate. A good example of the exposed situation is AMB which is a drug which is a drug that is associated with diverse serious adverse effects due to its toxicity when used in its free form, deoxycholate; in contrast, the colloidal dispersion, lipid complexes, and liposomes of AMB have fewer adverse effects and lower renal toxicity^26,28^. Given the high activity shown by compounds **4a–f** against *L. (V.) panamensis*, these compounds were selected for the evaluation of the therapeutic response in hamsters with experimental LC caused by *L. (V.) panamensis*.

None of the compounds **4a–f** showed hemolytic activity for huRBC with HA_50_ > 200 µg mL^−1^ values (Table 1). Regarding antiplasmodial activity, it can be observed that compounds **4a**, **4e** and **4f** showed high activity, with EC_50_ < 25 µg mL^−1^, and compounds **4b**, **4c** and **4d** showing moderate activity with EC_50_ > 25 µg mL^−1^. As expected, CQ, used as an assay positive control showed high activity (EC_50_ 3.4 µg mL^−1^). This observed antiplasmodial activity may be due to quinoline-like compounds’ capacity to affect the hemoglobin degradation pathway in the parasite or inhibit the synthesis of β-hematin (hemozoin pigmentation). When correlating hemolytic activity for huRBC with antiplasmodial activity, it was observed that compounds **4d** and **4f** had SI values > 11 (see Table 2). This results allowed for the selection of compounds **4e** and **4f** as hit compounds for validating its therapeutic effect in BALB/c mice experimentally infected with *P. berghei*.

**Table 2:** Effectiveness of treatment with compounds **4a–f**.

Treatment^*b*^	Result^a^ *n* (%)		
	Cure	Improvement	Failure
**4a**^*c*^	0 (0)	4 (80)	1(20)
**4b**	1 (20)	4 (60)	0 (0)
**4c**	1 (20)	4 (60)	0 (0)
**4d**	2 (40)	3 (60)	0 (0)
**4e**	0 (0)	4 (80)	1 (20)
**4f**	1 (20)	0 (0)	4 (80)
MA^*d*^	4 (80)	1 (20)	0 (0)

The data represent the number and percentages of hamsters, according to the result at the end of the study. ^a^At 3 months after the end of treatment. ^b^*n* = 5 animals per group. ^c^Via topical (40 mg) once/day/20 days. ^d^MA: meglumine antimoniate via IL (200 μg) twice/week/3 weeks. *cure*, 100% healing of the area and complete disappearance of the lesion; *improvement*, percentage of reduction around the lesion greater than 20%; *failure,* less than 20% of reduction of the area of lesion, or any increase in the size of the lesion.

### In vivo therapeutic response of compounds 4a–f in hamsters with experimental CL

The evolution of ulcers was monitored for 90 days after the end of the treatment. When the treatment was effective, the ulcerative lesions gradually regressed to complete healing (0.0 mm^2^) or reduced in size. On the contrary, when the treatment did not work, the size of the lesion increased. Treatment with 1% **4d** cream was the most effective, managing to cure of 40% of hamsters and producing improvement in the remaining of 60% the hamsters in the group (Table 2). For their part, 1% **4b** or **4c** cream managed to cure of 20% hamsters in each group and produced improvements in the 80% hamsters, while treatment with compound **4f** cured 20% hamsters and failed in the remaining in the 80% hamsters. On the other hand, 1% **4a** or **4e** cream produced improvement in the 80% hamsters and failures in the remaining hamster. As expected, MA treatment produced the highest percentage of cures, with the 80% of the hamsters cured (see Table 2).

The percentages of reduction (positive values) and increase of lesions (negative values) for each hamster in treatment groups are summarized in Table 3. Improvement percentages close to 80% were observed in Hamsters treated with compound **4d**, while in the four hamsters treated with compounds **4b** and **4c,** the improvement percentages were between 40.6 and 90.3%. In the compound **4a** group, the percentages of improvement ranged between 58.9 and 88.7%. Groups treated with compounds **4a** and **4f **failures in the treatment were evidenced by percentages of reduction of the lesion lower than 10% and some hamsters even showing negative values that corresponds to an increase in the lesion size (Table 3) (Figure S1 Supplementary Material).

**Table 3:** Effect of treatment with compounds **4a–f** on the size of the lesion.

Compounds/hamster #	Lesion size reduction (%)				
	**1**	**2**	**3**	**4**	**5**
**4a**	58.9	88.7	6.0	88.7	69.0
**4b**	58.8	90,3	56,6	100	76,4
**4c**	100	68.2	79.6	40.6	85.6
**4d**	79.1	100	100	78.1	79.5
**4e**	54.9	69.0	39.7	67.8	− 1.1^a^
**4f**	− 249	100	9.4	− 126	2.2
**MA**	100	100	78.0	100	100

The appearance of the lesions before treatment and at the end of the study in a representative hamster of each treatment (**4a–4f**) is shown in Figure S2 Supplementary Material.

Data shown the percentage of reduction of the lesion after 90 days of the end of treatment (PTD90). ^a^Negative value corresponds to an increase in the size instead a decrease.

The appearance of the lesions before treatment and at the end of the study in a representative hamster of each treatment (**4a–4f**) is shown in Figure S2 Supplementary Material.

No loss in average body weight of hamsters was observed during the study; therefore, no detrimental effect on hamster weight or toxic effects could be attributed to compound treatment. According to the weight of the animals at the beginning and during the study, no significant differences were observed in the groups of hamsters treated with compounds or MA. Likewise, no alterations were observed in the levels of ALT, BUN, and serum creatinine, which were measured 8 days after treatment with compounds **4a–f** and MA. No significant difference was observed between groups of treatments, and all measurements were inside normal value ranges (see Supplementary Material Table S2, Figure S3). These results suggest that liver and kidney function were not affected by treatment with compounds **4a–f**.

The results of this study are in line with the results of previous studies, this is evidenced in the similarity of the efficacy of the 2-alkyquinoline compounds such as 2-n-propylquinoline, 2-propenylquinoline and 2-trans-epoxypropylquinoline isolated from the bark. of the *Galipea longiflora* plant, which showed the same efficacy as the reference drug Glucantime, in reducing the size of the lesion caused by CL in a mouse model (BALB/c) infected with *L. amazonensis* and *L. venezuelensis*, without presenting symptoms of toxicity^14^. Likewise, the compounds obtained in this study were more active in vitro than the styrylquinolines reported by Loiseau et al. 2011, despite having the same structural core, which were evaluated against Leishmania donovani^75^. Additionally, these results also show similar activity with the styrylquinolines and the hybrid quinoline-hydrazone analogues evaluated against *L. (V.) panamensis* obtained by Cantero et al.^17^ and Coa et al.^70^, respectively.

### In vivo therapeutic response of 4e and 4f in BALB/c mice infected with *P. berghei*

None of the compounds (**4e** and **4f**) managed to control the infection caused by *P. berghei* under the evaluated scheme. On the contrary, parasitemia curves where on the rise until the end of the experiment. In general, the mean parasitemia levels rose daily during the 7 days of treatment. At day 1 and 2 post-treatment, parasitemia increased by 83% in group treated with compound **4f** in comparison with the end of the treatment while group treated with **4e** showed a slight decrease in parasitemia (5.1% in respects of the end of treatment). On the other hand, groups treated with CQ showed complete healing with elimination of parasitemia (Figure S4A).

During clinical evaluation, all the experimental groups showed typical signs of disease such as weight-loss, dehydration, isolation and bristly fur, which became more notorious after day 3 of treatment, ranging from mild to moderate levels. In groups treated with **4e**, one mouse had endpoint at day 6 and another two at the end of treatment because of the increase in parasitemia (15% in average) that affected animal wellbeing. Treatment with compound **4e** and **4f** resulted in a loss of weight equal to 16.2% in **4e** group and 1.6% in group **4f**, mice group treated with PBS had a weight loss of 10.4% while mice group treated with CQ showed no weight loss (Figure S4B). This weight reduction are associated with dehydration status and anemia due to infection persistence. In mice treated with **4e** and **4f** were observed alterations of ALT and BUN serum levels measured 2 days after the end of treatment and significant difference (p < 0.005) was observed between groups **4e** and **4f** in comparison to CQ or healthy mice (see Supplementary Material Table S3). These results suggest that liver and kidney function can be affected by treatment with compounds **4e** and **4f**.

During post-mortem anatomopathological evaluation all mice showed hepatomegaly and splenomegaly because of infection. Optimization of treatment scheme in terms of formulation, dosage and frequency of administration may be considered for enhancing the therapeutic response of the treatment. The oral route that was proposed because to treat malaria it is mandatory to use systematic routes of administration; this router was not effective in mice infected with *Plasmodium*. Therefore, for future work the intramuscular route could be evaluated. In the case of hamsters infected with *Leishmania*, the topical route did show effect and a reduction in the size of the lesions was evidenced but also complete cure.

### Computational analysis

**Figure 2:**
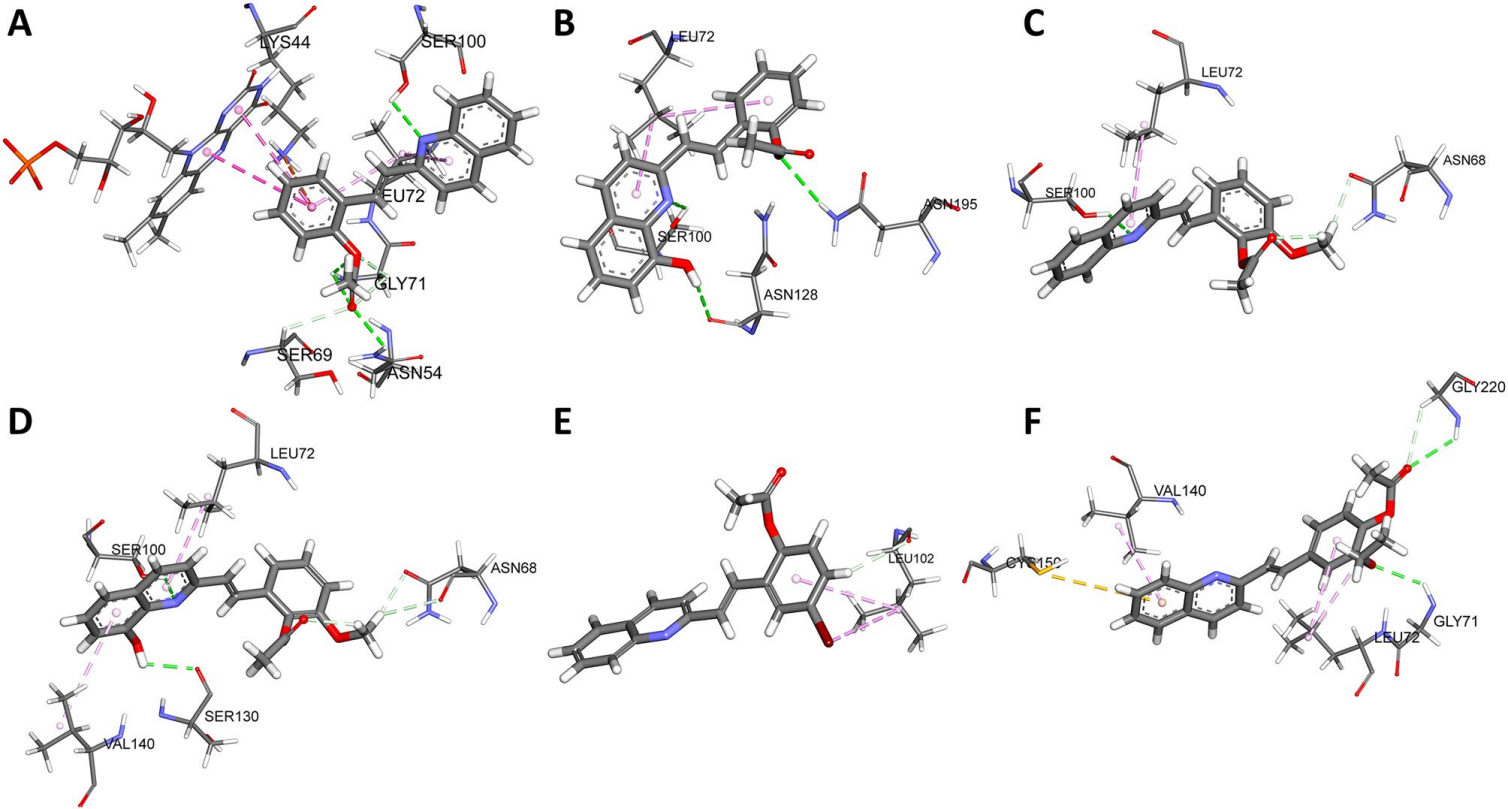
Docking analysis for ligands (**A**–**F**) 4a–f bound to LmDHODH. The surrounding amino acid residues in the binding pocket of LmDHODH within 3 Å.

According to results shown in Table 4, Fig. 2, and Tables S4 and S5 of supplementary information. The molecular docking experiments showed more favorable interactions, as well as ligand efficiency with *Lm*DHODH target. In general, the low *K*_*d*_ values suggest strong binding of the molecule to the protein. These analyses suggest that compounds **4a**–**f** exhibited promising activity against intracellular amastigotes of *L. (V.) panamensis*, due to these compounds forming a stable complex with each target studied. Table 4 also shows that **4b** and **4d** presented better interaction energies in 2-arylquinoline–*Lm*DHODH interactions. According to the experimental data, compounds **4b**–**d** were able to produce healing and improvement of the lesions in the hamsters after being treated. The interacting residues for both targets are summarized in Tables 5 and 6. Herein, it is possible to observe some residue differences in the binding modes of the active compounds.

**Table 4:** Molecular docking results for **4a–f** in the *Lm*DHODH and *Lm*TXNPx. Intermolecular docking energy values (*∆E*_*binding*_), *K*_*d*_ values, and calculated ligand efficiency (*LE*) for the *Lm*DHODH and *Lm*TXNPx complexes.

Compound	Docking results		Ligand efficiency			
	*Lm*DHODH	*Lm*TXNPx	*Lm*DHODH		*Lm*TXNPx	
	*∆E*_*binding*_ (kcal mol^−1^)	*∆E*_*binding*_ (kcal mol^−1^)	*K*_*d*_	*LE* (kcal mol^−1^)	*K*_*d*_	*LE* (kcal mol^−1^)
**4a**	− 7.7	− 7.6	2.27 $\times$ 10^–6^	0.35	2.69 $\times$ 10^–6^	0.35
**4b**	− 8.3	− 7.5	8.26 $\times$ 10^–7^	0.36	3.18 $\times$ 10^–6^	0.33
**4c**	− 7.6	− 7.1	2.69 $\times$ 10^–6^	0.32	6.26 $\times$ 10^–6^	0.30
**4d**	− 8.3	− 7.1	8.26 $\times$ 10^–7^	0.33	6.26 $\times$ 10^–6^	0.28
**4e**	− 7.7	− 7.4	2.27 $\times$ 10^–6^	0.33	3.77 $\times$ 10^–6^	0.32
**4f**	− 7.4	− 6.8	3.77 $\times$ 10^–6^	0.30	1.03 $\times$ 10^–5^	0.27

**Table 5:** ADMET molecular descriptors of compounds designed as leishmaniasis inhibitors.

Compound	*MW* (g mol^−1^)	*MR*	*LogP*	*HBA*	*HBD*	*TPSA* (Å^2^)	LR	GR	VR	PR	Synth. Acce
4a	289.33	88.61	3.88	3	0	39.19	0	0	0	0	2.53
4b	305.33	90.64	3.51	4	1	59.42	0	0	0	0	2.64
4c	319.35	95.1	3.84	4	0	48.42	0	0	0	0	2.78
4d	335.35	97.13	3.48	5	1	68.65	0	0	0	0	2.88
4e	368.22	96.31	4.46	3	0	39.19	0	0	0	0	2.68
4f.	333.38	99.91	4.16	4	0	48.42	0	0	0	0	2.66

^1^*LR* Lipinski rules, *G* : Ghose rules, *VR* Veber rules, *PR* Pfizer rules, *Synth. Acce*. synthetic accessibility. ^2^*MW* molecular weight, *MR* molar refractivity, *LogP* octanol/water partition coefficient, *HBA* hydrogen bond acceptor, *HBD* hydrogen bond donor, *TPSA* topological polar surface area, *RB* rotatable bond.

**Table 6:** Predicted binding free energies (kcal mol^−1^) and individual energy terms, calculated from molecular dynamics simulation through the MM/GBSA protocol for *Lm*DHODH complexes.

Compounds	Calculated free energy decomposition (kcal mol^−1^)			
	*ΔG*_*binding*_	*ΔE*_*vdW*_	*ΔE*_*elect*_	*ΔE*_*pot*_
**4a**	− 22.32 ± 0.13	− 33.10 ± 0.16	15.43 ± 0.07	− 17.66 ± 0.12
**4b**	− 25.73 ± 0.08	− 31.52 ± 0.09	10.02 ± 0.03	− 21.49 ± 0.07
**4c**	− 21.13 ± 0.13	− 29.29 ± 0.16	12.19 ± 0.08	− 17.09 ± 0.12
**4d**	− 30.05 ± 0.08	− 37.33 ± 0.08	11.88 ± 0.03	− 25.44 ± 0.08
**4e**	− 11.42 ± 0.12	− 16.73 ± 0.16	7.10 ± 0.06	− 9.62 ± 0.10
**4f**	− 18.59 ± 0.19	− 25.35 ± 0.20	10.43 ± 0.07	− 14.92 ± 0.17

*HBD* hydrogen bond donor, *TPSA* topological polar surface area, *RB* rotatable bond.

It should be noted that the molecular docking was performed with two crystals (*Lm*DHODH and *Lm*TXNPx) from *L. major*, but the biological experiments were performed with *L. (V.) panamensis*, although they belong to different organisms, but are part of the same Trypanosomatidae family of proteins and are highly conserved in the *Leishmania* and *Trypanosoma* genera^76^. Multiple alignments were performed with the Clustal Omega web server from orthologous sequences for *Lm*/*Lp*DHODH and *Lm*/*Lp*TXNPx (see multiple alignments Figures S9, S10 and S11 in supplementary information), where high identity and conservation is observed between the DHODH sequences for *L. major* (UnitProt ID: Q4QEW7) and *L. panamensis* (UnitProt ID: A0A088RLI8), with an identity percentage of 87%. For the case of TXNPx sequences for *L. major* (UnitProt ID: Q4QF76) and *L. panamensis* (UnitProt ID: A0A088RNK9), with an identity percentage of 84%. For a good percentage of identity between sequences it should always be higher than 50%^77^, which the homologous three-dimensional structure of *Lp*DHODH and *Lp*TXNPx will have conserved domains and a fairly conserved structural similarity at the alpha carbon positions with *Lm*DHODH and *Lm*TXNPx. In addition, previous studies have been used for molecular docking of *Lm*DHODH and *Lm*TXNPx crystals to represent the antileishmanial activity of specific compounds^17,38^.

In order to assess whether molecules can be selected as potential **4a–f** inhibitors, we calculated some pharmacokinetic properties (Table 5). These results were contrasted against Lipinski^78–80^, and Pfizer^76^ rules. If any of the compounds only satisfied two of the rules of Lipinski and Ghose, we took that compound as precautionary; if it satisfied only one rule, then this molecule is not a good candidate. Following Veber's rules, if a compound does not meet any of these parameters, then it is not a good drug candidate. Pfizer's toxicity rules were also taken into account—if any of our ligands did not meet these parameters, then it was not considered a good drug candidate. According to Table 5, it is observable that the candidates were within the range of expected values for the Lipinski's and Gelovani's parameters. Therefore, the compounds are expected to be active. However, the experimental evidence confirmed that the compounds are active through the dermal route. Additionally, the Boiled-egg model (see Figure S5 in supplementary information) was used to calculate the lipophilicity and polarity of these molecules. The results showed that all of the studied 2-arylquinolines are highly absorbable at the blood–brain barrier, while not being absorbable in the gastrointestinal tract (see Figure S6 in supplementary information). Finally, the ADMET properties suggested that these compounds may be safe compounds for use as leishmaniasis inhibitors (see Figure S5 in supplementary information).

The final snapshots of the molecular dynamics simulations are illustrated in Figure S7 (see supplementary information) Previous studies have shown that the FMN molecule plays a functional role in the active site of *Lm*DHODH, acting as a stabilizing cofactor of the active site and forming an aromatic box whose function is to stabilize the ligand^34,35,81^. The molecular simulation results showed differences in the binding and interaction of compounds **4a**, **4b**, **4c**, **4d**, and **4f** with the main binding site (BP1); see Figures S7 and S8 in supplementary information).The results also show that, throughout the simulation trajectory, compound **4a** remained stable in this original binding site (where the catalytic function of *Lm*DHODH is found, regions S1–S5); interacting in a stable way with FMN and representing a 10% interaction throughout the molecular dynamics simulation (see Figure S9 in supplementary information). On the other hand, compounds **4b**, **4c**, **4d**, and **4f** were stably located in a position close to the binding site of *Lm*DHODH (BP2 site), with a low percentage of participation of the residues in the regions S1–S5; this fluctuation space made a null interaction with the FMN cofactor, (see Figure S7 and S8 in supplementary information). For the case of compound **4e** at 24 ns of the trajectory, it left the *Lm*DHODH binding site and remained free in the solvated medium (see Figure S7 and S8 in supplementary information).

Molecular dynamics simulations showed that LmDHODH residues directly interacted with the ligands (**4a–f**). The most frequent LmDHODH residues are illustrated in Figure S9 (see in supplementary information). Additionally, the potential inhibitors evaluated here interacted with the before-mentioned pockets (BP1 and BP2) through electrostatic and hydrophobic interactions. In the case of compound 4a, it showed interactions with the S1–S4 regions, with the residues Gly71, Leu72, Ser100, Ser130, Gln139, Asn128, Asn195, Phe218, Gly198, Ser196, and Ile197 (see Figure S9 in supplementary information). These coincide with those previously reported by the scientific community, which are the S1, S2, S3, and S4 sites, thus validating the protocol used in this work^34,35,38,82^. For compounds 4b, 4c, 4d, and 4f, a low percentage was shown, with the only interaction region being S2, involving residues Leu102, Leu72, Val140, Asn107, Cys150, Asn107, Val140, and Ser100; the other residue interactions were distributed at the BP2 binding site. In the case of compound 4e, there was no stable interaction at the main binding site, causing the escape of this compound into the solvated medium during the molecular simulation. These results document that compounds 4a–f are reversible inhibitors of LmDHODH^81^.

The binding free energy was computed after the MD simulation, considering the last 70 ns for all of the complexes; the results are given in Table 4. Compound 4d had a binding free energy of − 30.05 kcal mol^−1^ with the LmDHODH enzyme, while compound 4b showed a comparable binding free energy of − 25.73 kcal mol^−1^. In the case of compounds 4a and 4c, they showed relatively higher binding energy, with values of − 22.32 kcal mol^−1^ and − 21.13 kcal mol^−1^, respectively. Compounds 4e and 4f had the highest binding energy values (− 11.42 kcal mol^−1^ and − 18.59 kcal mol^−1^, respectively), indicating the low stability of these compounds at the LmDHODH binding site; (see Figures S8 and S9 in supplementary information). The results obtained from free energy (see Table 6) calculations also demonstrated that compound 4a had a higher binding energy than compound 4d, with an absolute difference of 7.73 kcal mol^−1^. This difference was due to the interaction with the cofactor flavin mononucleotide. In particular, the 4a compound had better activity at both the experimental and in silico levels. The results of this study constitute a significant advance to be applied in the search and development of new treatment alternatives for cutaneous leishmaniasis (CL)^83^. A previous work reported the experimental properties of eight similar compounds in terms of the base structure were studied, also giving good results from the experimental and theoretical perspective, showing excellent in vitro activity against intracellular amastigotes of *L. panamensis*^17^.

## Conclusions

Compounds **4a–f** showed promising potential as leishmanicidal products for treating cutaneous leishmaniasis caused by *L. (V.) panamensis*. The 1% cream formulations containing compounds **4b–d** were able to heal and improve the CL lesions in hamsters after topical treatment without signs of local or systemic toxicity. Compounds **4a–f** showed in vitro antiplasmodial activity and none of the tested compounds (**4e** and **4f**) managed to control infection by *P. berghei* under the tested scheme. Molecular dynamics simulations and free energy studies revealed that the compound **4a** has a preferential interaction with the cofactor flavin mononucleotide, suggesting better activity at the in silico level, which was confirmed by our experimental results. A computational docking study showed that molecules **4b** and **4d** present better interaction energies in 2-arylquinoline–*Lm*DHODH interactions, in agreement with the experimental data regarding the treatment of hamster lesions. Finally, we predicted that the synthesized 2-arylquinolines are absorbable at the blood–brain barrier, but they have no action in the gastrointestinal tract. Nevertheless, further studies are needed to validate the usefulness of these cream formulations for treating CL caused by other *Leishmania* species. Will be also necessary to evaluate other formulation containing an increased amount of each active compound.

### Permission to reuse and copyright

Figures, tables, and images will be published under a Creative Commons CC-BY licence and permission must be obtained for use of copyrighted material from other sources (including re-published/adapted/modified/partial figures and images from the internet). It is the responsibility of the authors to acquire the licenses, to follow any citation instructions requested by third-party rights holders, and cover any supplementary charges.

### Supplementary Information

Supplementary Information.

## Data Availability

All data generated or analysed during this study are included in this published article [and its supplementary information files.
