# Supplementary material for: Screening of the antileishmanial and antiplasmodial potential of synthetic 2-arylquinoline analogs
Source: Sci Rep. 2023 Oct 16;13:17523. doi: 10.1038/s41598-023-43805-4 (PMC10579228; doi:10.1038/s41598-023-43805-4)
Supplement: Supplementary file 1 — Supplementary Information. [file 41598_2023_43805_MOESM1_ESM.docx]

Supplementary Material


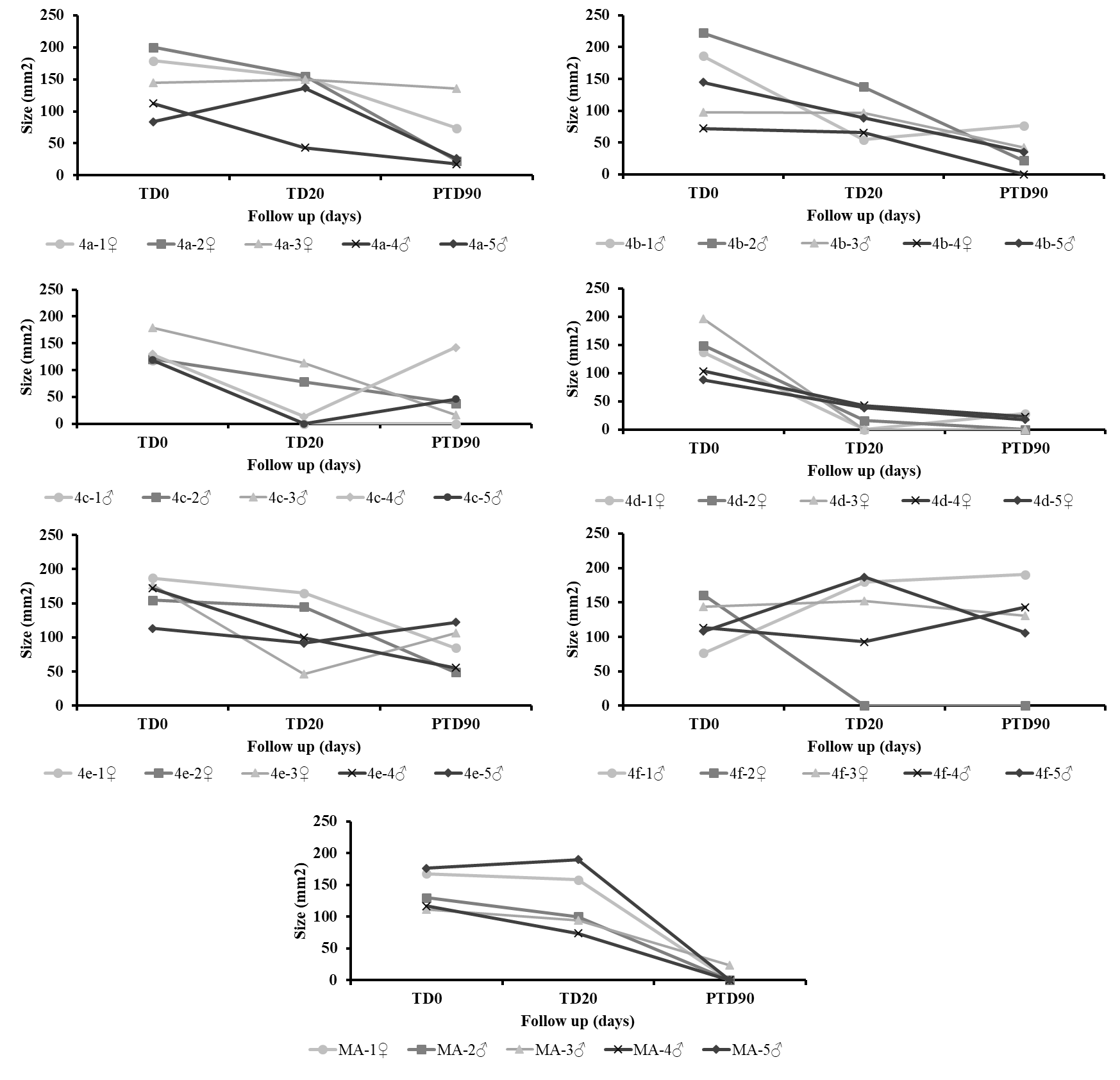


**Figure. S1 Therapeutic response of hamsters with Cutaneous Leishmaniasis (LC) to the treatment with (4a-4f) vs Meglumine Antimoniate.** Response was evaluated in hamsters treated with 1% cream formulation of **4a-4f** at dose of 40 mg/day (topic) vs. intralesional meglumine antimoniate (MA). Lines represent the evolution of lesion size in mm^2^ before and after treatment follow up. TD0: Before treatment; TD20 last day of treatment; PTD30: day 30 after the end of treatment; PTD60: day 60 after treatment ended; PTD90: day 90 after the end of treatment.


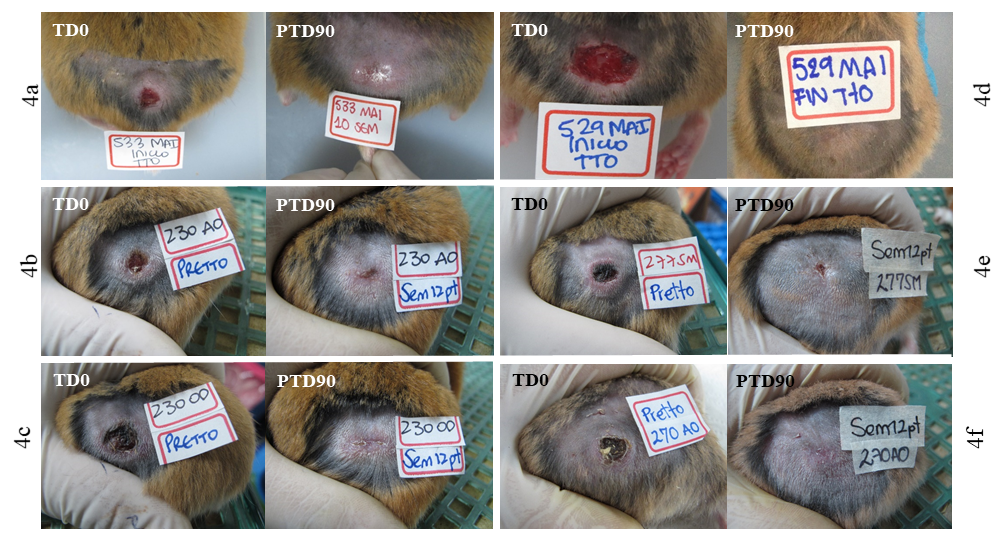


Figure S2 Treatment progress of cutaneous leishmaniasis (CL) caused by *L. (V.) panamensis* in hamsters treated with compounds 4a–f, before treatment (TD0) and on day 90 after the end of treatment (PTD90). Observe the decrease in the size of the lesion at three months after treatment, with respect to that at the beginning of the treatment

Figure S3 C**hanges in the weight of hamsters infected with** L (V.) panamensis after treatment with 1% 4a-4f cream formulation***.* The** x**-axis corresponds to time points of study, while the** y**-axis shows the weight** average **of hamsters from before treatment until the end of the study. Data represents median of weight ± standard deviation. TD0: before treatment; TD20: last day of treatment; PTD30:** day 30 after the end of treatment; PTD60: day 60 after treatment ended; PTD90: day 90 after the end of treatment.


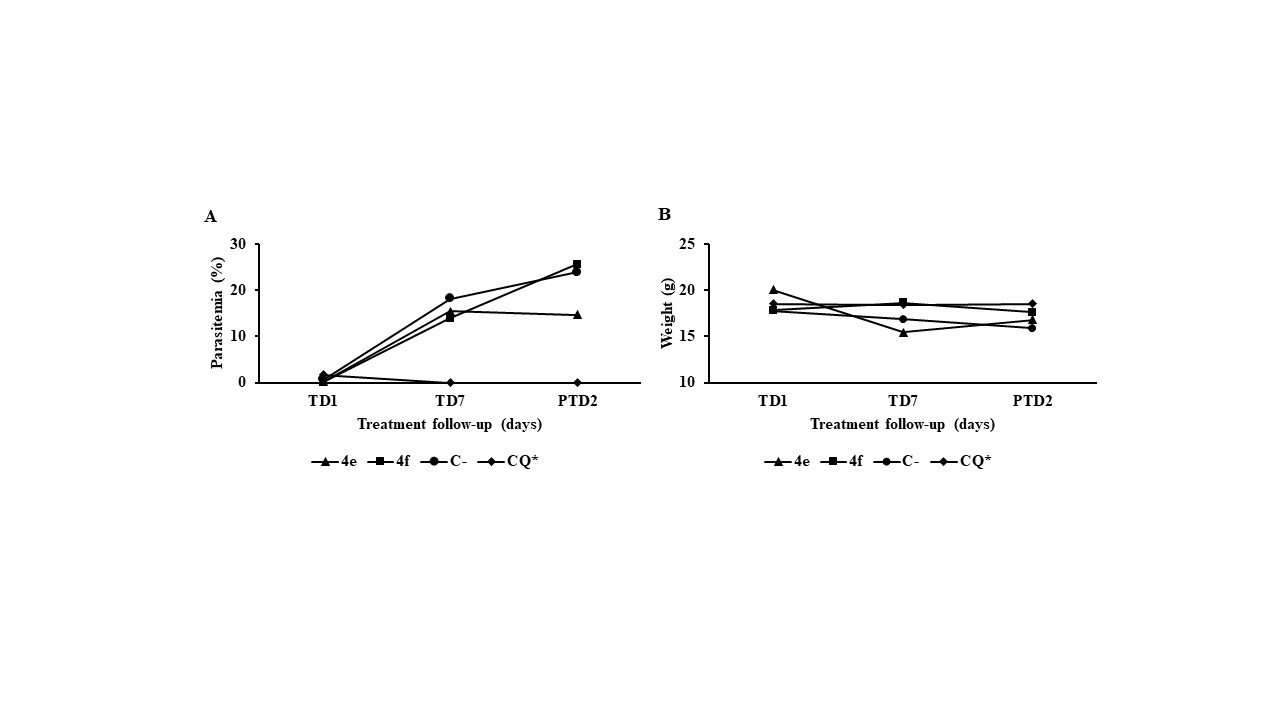


**Figure S4.** Effect of treatment with 4e and 4f on the course of *P. berghei* infection in BALB/c mice. The graph shows the parasite load (A) and the weight (B) at the beginning of the treatment (TD0), at the end of the treatment (TD7) and at the end of the study, which corresponded to day 2 after the end of the treatment (PTD2). *The last day of treatment with CQ was day 4.

**Table S1.** Canonical SMILES and formula of compounds designed as leishmaniasis inhibitors.

| Molecule | Canonical SMILES | Formula |
| --- | --- | --- |
| **4a** | CC(=O)Oc1ccccc1/C=C/c1ccc2c(n1)cccc2 | C_19_H_15_NO_2_ |
| **4b** | CC(=O)Oc1ccccc1/C=C/c1ccc2c(n1)c(O)ccc2 | C_19_H_15_NO_3_ |
| **4c** | COc1cccc(c1OC(=O)C)/C=C/c1ccc2c(n1)cccc2 | C_20_H_17_NO_3_ |
| **4d** | COc1cccc(c1OC(=O)C)/C=C/c1ccc2c(n1)c(O)ccc2 | C_20_H_17_NO_4_ |
| **4e** | CC(=O)Oc1ccc(cc1/C=C/c1ccc2c(n1)cccc2)Br | C_19_H_14_BrNO_2_ |
| **4f** | CCOc1cc(/C=C/c2ccc3c(n2)cccc3)ccc1OC(=O)C | C_21_H_19_NO_3_ |

**Table S2.** Toxicological parameters measured in the *in vivo* assay in hamsters (**4a-f**)

| **Compounds** | **ALT (UI)**  **R.V: 22-128** | | **Creatinine (mg/dL)**  **R.V 0.3-1.0** | | **BUN (mg/dL)**  **R.V 25-55** | |
| --- | --- | --- | --- | --- | --- | --- |
|  | **TD0** | **TD8** | **TD0** | **TD8** | **TD0** | **TD8** |
| **4a** | 62.7+7.7 | 64.5+11.7 | 0.35+0.1 | 0.3+0.1 | 31.5+3.7 | 39.0+1.8 |
| **4b** | 55.3+5.8 | 69.3+10.1 | 0.3+0 | 0.3+0 | 27.3+0.6 | 30.0+2.0 |
| **4c** | 78.7+8.6 | 60.8+6.9 | 0.4+0.1 | 0.4+0.1 | 26.4+5.8 | 36.9+3.1 |
| **4d** | 57.4+2.3 | 71.0+14.1 | 0.35+0.1 | 0.4+0.1 | 31.7+1.2 | 33.0+4.0 |
| **4e** | 62.0+8.8 | 63.7+11.7 | 0.4+0.1 | 0.4+0.1 | 29.9+2.1 | 35.4+5.5 |
| **4f** | 76.8+11.2 | 51.0+8.1 | 0.4+0.05 | 0.5+0.05 | 34.3+2.3 | 34.0+6.2 |
| **MA** | 72.3+16.6 | 74.5+10.2 | 0.43+0.08 | 0.4+0.1 | 38.6+15.8 | 42.5+9.7 |

Data represent the mean values + SD. **TD0**: Before Treatment; **TD8**: 8 days of Treatment. **MA**: meglumine antimoniate

**Table S3.** Toxicological parameters measured in the *in vivo* assay in mice (**4e-f**)

| **Compounds** | **ALT (UI)**  **R.V: 17-77** | | **Creatinine (mg/dL)**  **R.V 0.2-0.9** | | **BUN (mg/dL)**  **R.V 8-33** | |
| --- | --- | --- | --- | --- | --- | --- |
|  | **TD0** | **PTD2** | **TD0** | **PTD2** | **TD0** | **PTD2** |
| **4e** | 50.0+5.2 | 357.0+39.3 | 0.4+0 | 0.7+0.1 | 11.8+1.6 | 117+6.5 |
| **4f** | 56.1+3.4 | 216.0+28.1 | 0.5+0 | 0.4+0.1 | 12.6+2.9 | 86.0+1.0 |
| **CQ** | 70.3+6.3 | 34.0+1.7 | 0.4+0.1 | 0.4+0.1 | 10.0+2.1 | 20.5+5.8 |
| **Healthy** | 57.4+5.9 | 77.0+4.6 | 0.4+0.1 | 0.7+0.1 | 31.7+1.2 | 29.4+2.5 |

Data represent the mean values + SD. TD0: Before Treatment; PTD2: 2 days after the end of treatment. CQ: Chloroquine

Table S4. Molecular docking results for 4a–f in the *Lm*DHODH. The interacting residues within a radius of 3Å and the most relevant hydrogen bonds in the complexes are shown.

| ***Lm*DHODH** | | |
| --- | --- | --- |
| **Compound** | **H-bonds interaction** | **Residue interaction** |
| **4a** | - | asn128, asn54, asn68, gly101, gly71, leu102, leu72, met70, ser100, ser103, ser130, ser69, val140 |
| **4b** | - | asn128, asn195, asn68, gly101, gly71, leu102, leu129, leu72, lys44, met70, ser100, ser130, ser196, val140 |
| **4c** | asn68 | asn128, asn195, asn68, gln139, gly101, gly220, leu102, leu72, lys44, met70, ser100, ser130, ser196, val140 |
| **4d** | asn68 | asn128, asn68, gln139, gly101, gly220, leu102, leu129, leu72, lys44, met70, ser100, ser130, ser196, ser69, val140 |
| **4e** | fmn | asn107, asn128, asn68, gln139, gly101, gly220, leu102, leu129, leu72, ser100, ser130, ser196, val140, fmn |
| **4f** | - | asn107, asn128, asn195, asn68, cys150, gly101, gly220, gly71, leu102, leu129, leu72, lys44, met70, ser100, ser103, ser130, ser196, ser69, val140 |

Table S5. Molecular docking results for 4a–f in the *Lm*TXNPx. The interacting residues within a radius of 3Å and the most relevant hydrogen bonds in the complexes are shown.

| ***Lm*TXNPx** | | |
| --- | --- | --- |
| **Compound** | **H-bonds interaction** | **Residue interaction** |
| **4a** | val172, leu46, met147, phe50 | arg128, arg140, cys52, glu167, leu46, met147, phe50, pro148, pro186, pro45, thr49, val166, val172, val51 |
| **4b** | val172, leu46, met147, phe50 | arg128, arg140, cys52, leu46, met147, phe50, pro148, pro186, pro45, thr49, val166, val172, val51 |
| **4c** | pro148, thr49 | arg128, arg140, asn9, asp146, leu46, met147, phe50, pro148, pro45, thr49, tyr127, val125, val166, val172, val51 |
| **4d** | val172 | arg128, asn9, asp146, cys52, leu46, met147, phe50, pro148, pro186, pro45, thr49, val172, val51 |
| **4e** | pro148, val125, phe50, met147 | arg128, cys52, glu171, gly170, leu46, met147, phe50, pro148, pro186, pro45, thr49, val125, val166, val172, val51 |
| **4f** | met147, phe50 | arg128, asn9, cys52, gln123, glu120, leu46, met147, phe50, pro148, pro186, pro45, ser122, thr49, val125, val172, val51 |


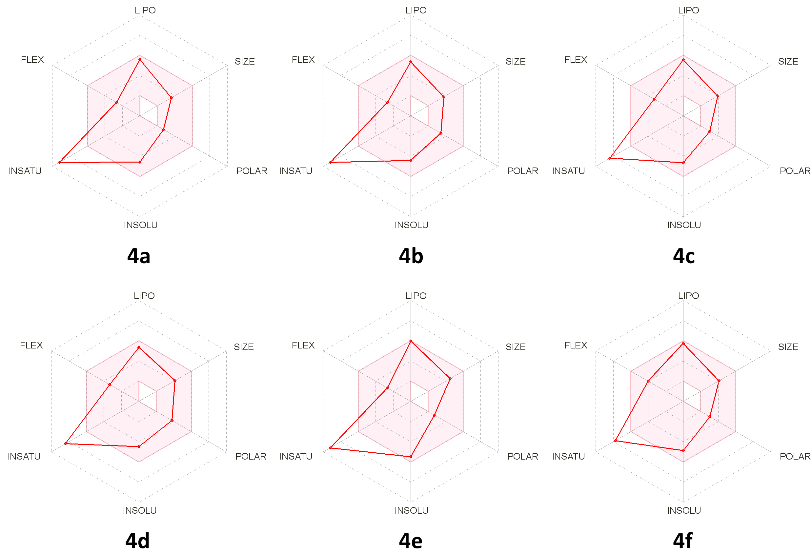


**Figure S5.** Analysis of six physicochemical properties (lipophilicity, size, polarity, solubility, flexibility, and saturation) using bioavailability radar plot representations. The shaded area represents the range of properties to be considered drug-like. The black line represents the properties of the test molecules.

**
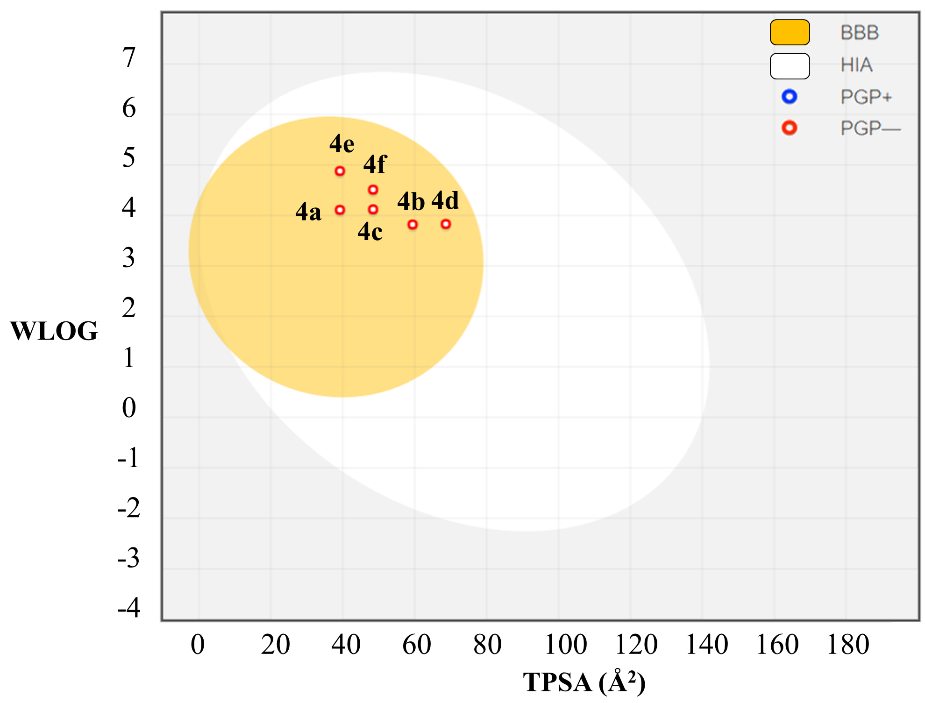
**

**Figure S6.** Predictive human intestinal absorption (HIA) model and blood–brain barrier permeation (BBB) method (boiled-egg plot) of the six considered compounds (**4a–f**).


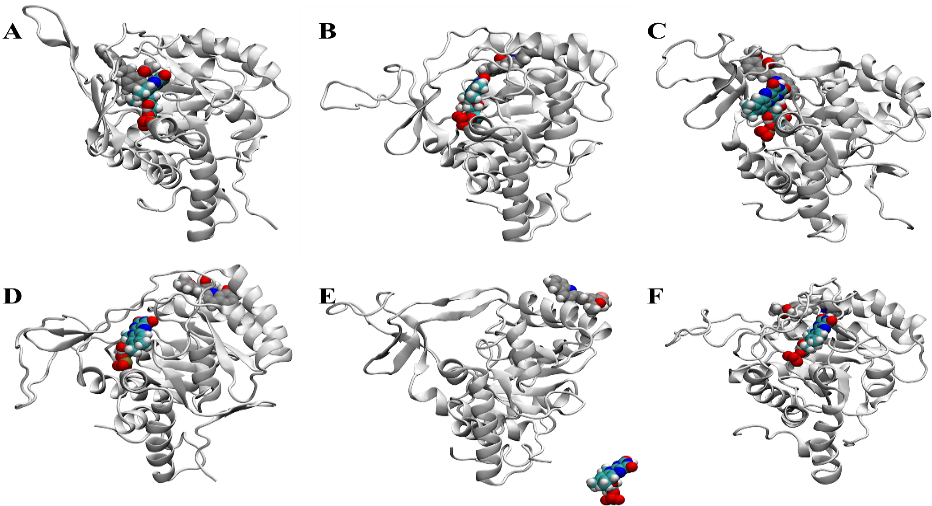


Figure S7. Last snapshot (taken at 70 ns) of molecular dynamics simulations of *Lm*DHODH in complex with FMN molecule and compounds 4a–f at BP1 site. *Lm*DHODH is represented in white, FMN is coloured cyan, and 4a–f are coloured grey.


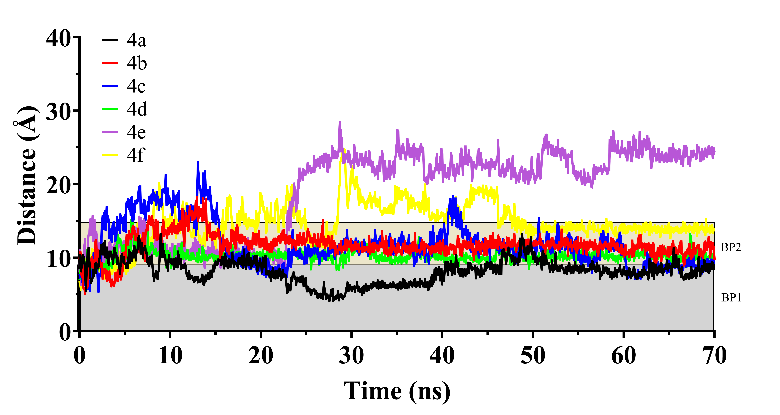


Figure S8. Distance (as a function of simulation time) between the centre of mass of the *Lm*DHODH protein and centre of mass compounds 4a–f. BP1 and BP2 correspond to *Lm*DHODH binding sites one and two, respectively.


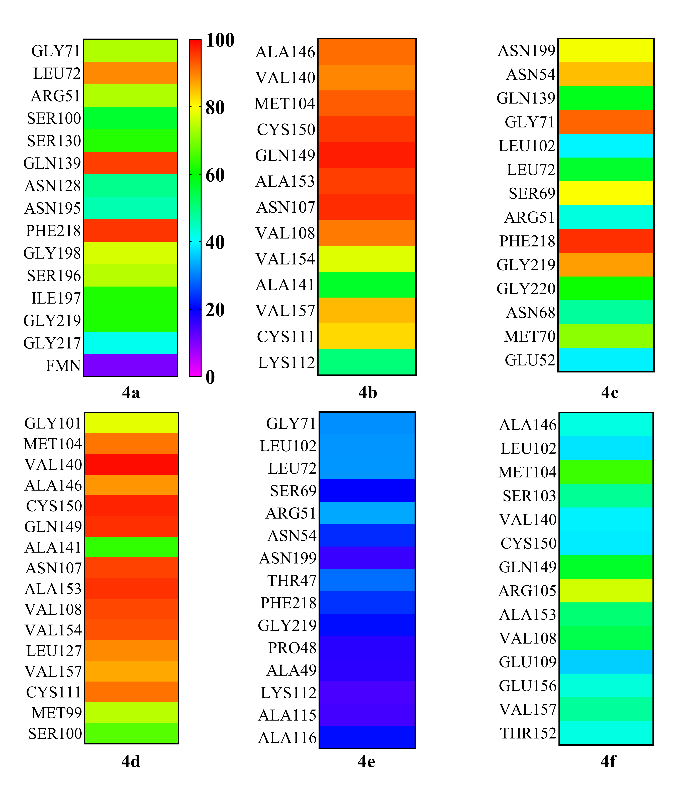


Figure S9. Percentage rate of interaction between *Lm*DHODH residues and compounds 4a–f.


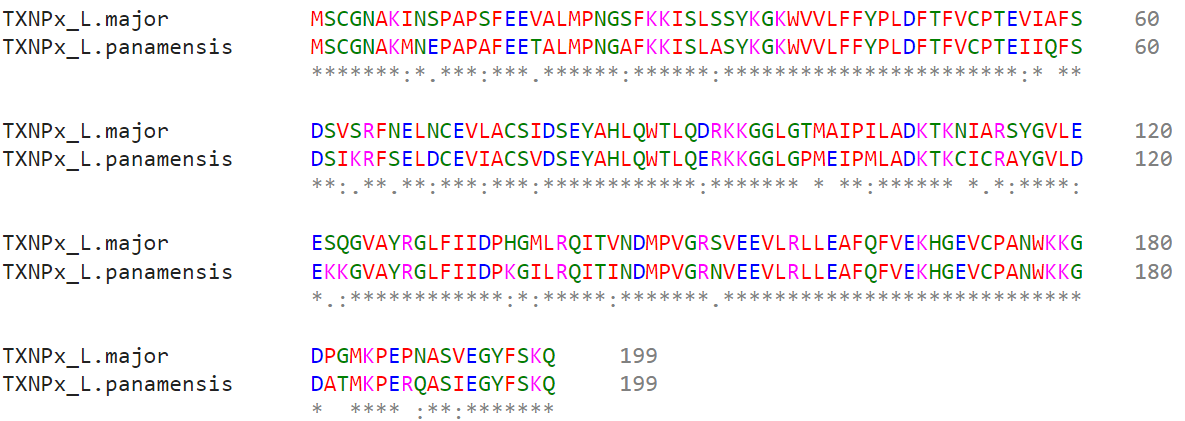


**Figure. S10** Alignment between *Lm*TXNPx and *Lp*TXNPx protein sequences.


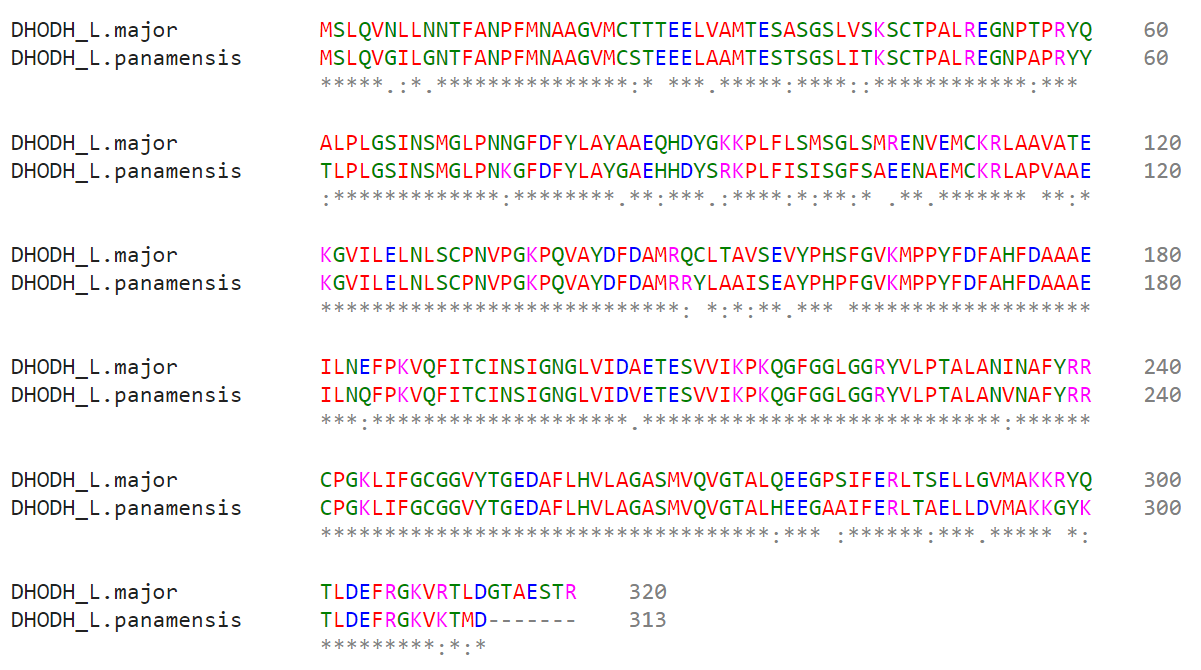


**Figure S11**- Alignment between *Lm*DHODH and *Lp*DHODH protein sequences.
